# Selection of Functional Glycoforms in Anti-SARS-CoV‑2 Human IgG1 Monoclonal Antibodies by FcγRIIIa Affinity Chromatography and Mass Spectrometry

**DOI:** 10.1021/acs.jmedchem.6c00166

**Published:** 2026-04-04

**Authors:** Barbara Oliviero, Sunil Kumar, Daniela Conteianni, Gaia Donetti, Antonella Cerino, Antonino Samuele Iraci, Alessia La Gaipa, Sabrina Ottolini, Sara Tengattini, Gabriella Massolini, Irene Cassaniti, Josè Camilla Sammartino, Dalila Mele, Fausto Baldanti, Federico Forneris, Silvia Faravelli, Claudia Scotti, Greta Pessino, Maristella Maggi, Stefania Mantovani, Caterina Temporini, Mario U. Mondelli, Marco Terreni

**Affiliations:** † Department of Translational and Clinical Research, Division of Molecular Medicine, Laboratory of Clinical Immunology, 18631 Fondazione IRCCS Policlinico San Matteo, 27100 Pavia, Italy; ‡ Department of Drug Science, 19001 University of Pavia, 27100 Pavia, Italy; § Department of Clinical-Surgical, Diagnostic and Pediatric Sciences, Università degli Studi di Pavia, 27100 Pavia, Italy; ∥ Department of Microbiology and Virology, Fondazione IRCCS Policlinico San Matteo, 27100 Pavia, Italy; ⊥ Department of Biology and Biotechnology, The Armenise-Harvard Laboratory of Structural Biology, University of Pavia, 27100 Pavia, Italy; # Department of Molecular Medicine, Unit of Immunology and General Pathology, University of Pavia, 27100 Pavia, Italy; ¶ Department of Internal Medicine and Therapeutics, University of Pavia, 27100 Pavia, Italy

## Abstract

Monoclonal antibodies activate immune effector cells through Fc–Fcγ receptor interactions, which are strongly influenced by Fc region glycosylation. In this study, three anti-SARS-CoV-2 IgG1 human monoclonal antibodies (hmAbs1–3) derived from the B-cell clones of vaccinated (hmAbs1–2) and convalescent (hmAb3) individuals were investigated, with hmAb3 showing the strongest neutralization activity. Glycoform analysis revealed that hmAb1 predominantly contained ∼60% combined G1F and G2F glycoforms, while hmAb2 consisted of ∼63% G1F, G2F, and G2FS1. In contrast, hmAb3 displayed the greatest glycan diversity with ∼75% comprising G1F, G2F, G2FS1, and G2S1. FcγRIIIa affinity chromatography separated hmAb glycoforms based on receptor affinity, yielding five distinct peaks. Antibody-dependent cellular cytotoxicity (ADCC) assays showed that hmAb3 exhibited the highest activity. Further evaluation of individual hmAb1 fractions collected from the FcγRIIIa affinity column demonstrated a clear correlation between glycosylation patterns and ADCC activity, highlighting the critical roles of Fc galactosylation and sialylation in modulating the effector function.

## Graphical abstract

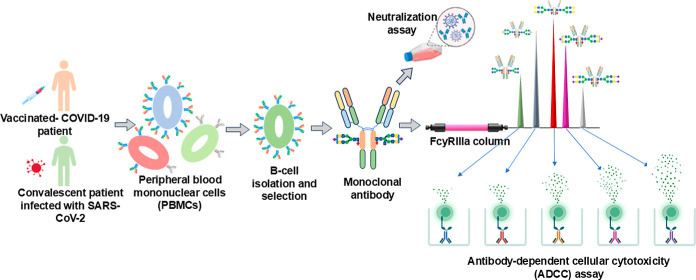

## 1 Introduction

Coronavirus disease 2019 (COVID-19), declared pandemic by the World Health Organization in March 2020, has resulted in over 777 million cases and more than 7 million deaths as of May 2025 ^1^ and highlighted the need for multidisciplinary approaches for the development of efficient biotherapeutic drugs for this and other viral respiratory diseases. To combat the infection, the scientific community has made remarkable progress in developing vaccines and therapeutic biomolecules over the years. During viral infections, the adaptive immune responses are mediated by a broad polyclonal antibody repertoire that plays a pivotal role through their multiple functions. Primarily, the antibodies (Abs) block virus entry in the host cells (neutralizing activity) through targeting specific epitopes. In severe acute respiratory syndrome coronavirus-2 (SARS-CoV-2) infection, neutralizing Abs target the virus Spike protein in the receptor-binding domain (RBD) that allows the virus to enter lung epithelial cells by interacting with the membrane-expressed angiotensin-converting enzyme 2 (ACE2) receptor.^2,3^ SARS-CoV-2 has evolved, developing several mutations and consequently numerous variants that altered virus functional properties such as its infectivity, virulence, transmissibility, and interactions with host immunity.^4,5^ During the pandemic, treatment approaches harnessed monoclonal antibodies (mAbs) to mimic and refine the neutralizing ability of natural Abs. Several therapeutic mAbs isolated from convalescent patients or immunized animals and including the REGN-COV2 cocktail (casirivimab and imdevimab), bamlanivimab, and bebtelovimab^6–8^ were developed by biopharmaceutical companies to treat early-stage COVID-19.^9^ However, these mAbs cannot be considered to be part of the natural immune response.

In addition to neutralizing activity, the efficiency of mAbs in infection control depends also on their ability to form immune complexes with viral molecules, inducing their clearance. Indeed, the crystallizable fragment (Fc) region of mAbs triggers the immune cell response by interacting with Fcγ receptors (FcγRs) expressed on several immune effector cells such as natural killer (NK) cells and phagocytes.^5^ In particular, NK cell activation via FcγRIIIa (CD16) is not induced by individual Fc–FcR interactions but occurs when immune complexes, composed of multiple antibodies bound to repeated epitopes on a multivalent antigen, bring several FcγRIIIa molecules into close proximity, promoting receptor cross-linking and clustering. This spatial organization triggers phosphorylation of the immunoreceptor tyrosine-based activation motif in the associated signaling adaptor chains (FcεRIγ and CD3ζ), leading to the release of perforin- and granzyme B-containing cytotoxic granules and ultimately resulting in antibody-dependent cellular cytotoxicity (ADCC).^10–12^

The mAb-Fc-effector function has been largely investigated in cancer but could also play a relevant role in the immune defense against viral infection such as SARS-CoV-2.^4,5^ The oligosaccharide composition of the Fc region plays a pivotal role in modulating interactions with FcγRIIIa, directly influencing ADCC and the overall therapeutic efficacy of mAbs,^13,14^ as observed during COVID-19. Indeed, the glycosylation pattern of the Fc could influence the severity of disease, being the severe COVID-19 patients but not those with mild symptoms featured by a lower or absent fucosylation in the Fc of anti-Spike IgG that induced proinflammatory cytokine release and led to the acute phase responses.^15,16^

IgG Abs possess a conserved N-linked glycosylation site at asparagine 297 (N297) within the Fc region.^17^ The glycan structures attached at this site can differ in composition ranging from high-mannose and hybrid to complex-type glycans, depending on the host cell line used for expression (Figure 1). Recombinant therapeutic mAbs are commonly produced using mammalian cell lines such as Chinese Hamster Ovary (CHO), HEK293, or mouse myeloma cells that typically generate complex glycoforms, with a dominant presence of the agalactosylated glycoforms (G0F).^18^ Alongside G0F, lower levels of mono- and digalactosylated (G1F and G2F, respectively) and sialylated glycoforms are also present, but in much smaller proportions. In contrast, endogenous IgG Abs from healthy human individuals exhibit a more balanced glycosylation profile. The predominant glycoforms include G0F, G1F, and G2F, typically found in approximately 35%, 35%, and 15% of total IgG molecules, respectively.^18^ Additionally, over 90% of human IgG N-glycans carry a core fucose residue attached to the innermost N-acetylglucosamine (GlcNAc) of the glycan core structure. Other glycan features such as bisected GlcNAc (bi-GlcNAc) and terminal sialic acid are present in 15–20% of IgG antibodies in healthy individuals.^19^

**Figure 1:**
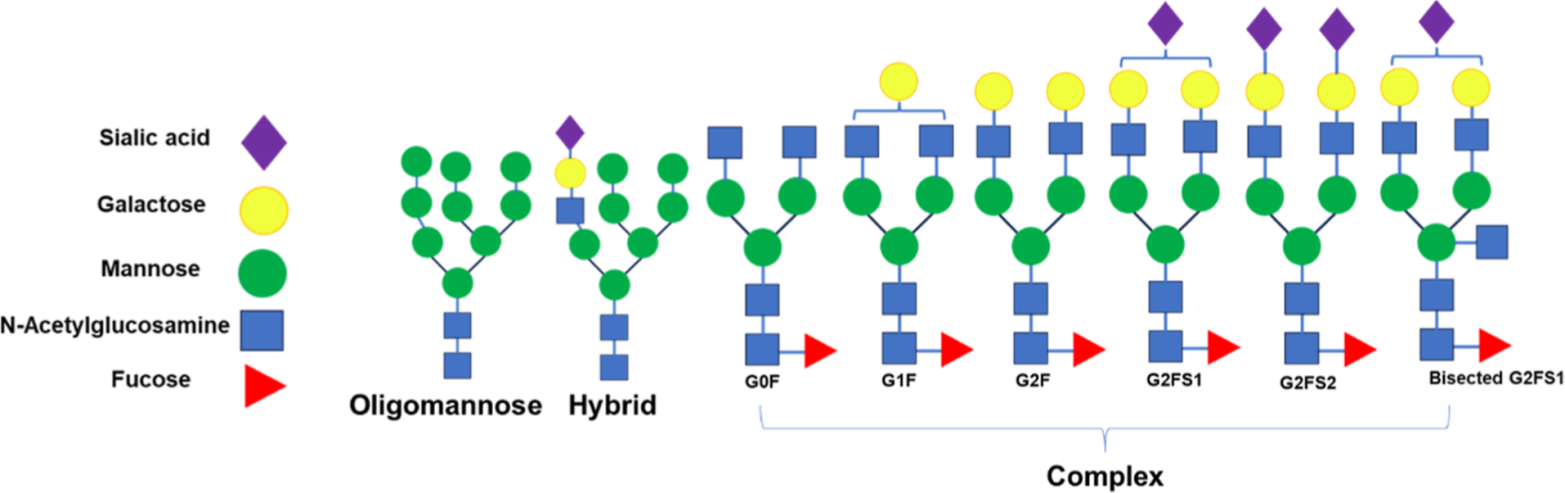
Schematic representation of N-glycan structures.

The presence or absence of core fucose on the Fc region of mAbs has a well-established impact on their biological activity, particularly on ADCC. Specifically, the lack of core fucose significantly enhances the binding affinity of the Fc region to FcγRIIIa on immune effector cells, thereby amplifying ADCC responses.^20^ In addition to fucosylation, the role of galactosylation and sialylation in modulating ADCC activity has also been investigated, although findings have been somewhat inconsistent. Some studies have reported that increased galactose content on Fc glycans correlates with enhanced ADCC activity.^19,21^ However, other investigators have observed either no significant effect or even contradictory results, highlighting that the impact of galactosylation may be context-dependent.^19^ Similarly, the influence of sialylation on ADCC and Fc receptor interactions remains unclear. Most studies suggest that terminal sialic acid residues (Neu5Ac) have minimal impact on ADCC activity as well as on binding to FcγRI and FcγRIIIa. However, sialylation may result in a slight enhancement of binding to FcγRIIa.^19,21^ The ambiguity in understanding glycan-mediated effects may stem from the high structural diversity of IgG glycans and their dependence on the host expression system. For instance, human endogenous Fc-glycans predominantly carry sialic acid in an α2,6-linkage with galactose (Neu5Ac-α2,6-Gal), whereas CHO cells—commonly used for mAbs production—lack the ability to synthesize Neu5Ac-α2,6-Gal and instead produce glycans with only α2,3-linked sialic acid residues. This distinction is functionally significant.^22^ Fc glycans with Neu5Ac-α2,6-Gal enable the Fc domain to adopt both open and closed conformations, thereby enhancing its conformational flexibility and introducing new functional characteristics. Thus, glycoengineering approaches have provided compelling evidence that α2,6-sialylated glycans can enhance the therapeutic efficacy of mAbs by improving ADCC starting from the anticancer mAb trastuzumab.^17^ However, the authors declare that more studies are required to clearly define the optimal glycan structure for ADCC targeting NK cells and/or other effector cells. Similarly, glycoengineered rituximab containing Neu5Ac-α2,6-Gal exhibited significantly higher ADCC activity compared to its α2,3-sialylated counterpart.^23^

These considerations motivated us to investigate the interactions between the Fcγ receptors and therapeutic mAbs. Surface plasmon resonance (SPR) bioassay is commonly used to carry out these studies^23,24^ but lacks the molecular resolution necessary to distinguish the individual contributions of different IgG glycoforms to FcγR binding.^20^ Recently, FcγRIIIa-based affinity liquid chromatography was introduced to study the interaction between Fc glycoforms and FcγRIIIa in vitro. Lippold et al. demonstrated chromatographic resolution of fucosylated and afucosylated antibody species using a custom-made FcγRIIIa receptor-based affinity column, in which the ligand was the FcγRIIIa receptor protein expressed in mammalian cells, thereby preserving native glycosylation.^25^ Conversely, the ligand employed in the only commercially available and widely used TSKgel FcR-IIIA-NPR affinity column is a modified recombinant mutant form of the human FcγRIIIa receptor. This recombinant ligand is expressed in an *E. coli* system, which inherently produces proteins lacking N-linked glycosylation, but the mutation introduced allowed us to maintain a similar folding compared with the natural glycosylated receptor. As a result, the FcγRIIIa ligand immobilized on the column has a molecular weight of approximately 20 kDa and does not contain glycan structures, including the N162 glycan known to influence IgG–FcγRIIIa interactions. Following expression and purification, the recombinant FcγRIIIa is covalently immobilized onto nonporous polymethacrylate beads that constitute the column’s stationary phase. Importantly, this ligand contains eight amino acid substitutions relative to the wild-type FcγRIIIa, introduced to enhance structural stability, as previously reported.^26^ Interestingly, the structural suitability of the nonglycosylated FcγRIIIa variant for studying IgG interactions has been well validated. Kiyoshi et al. demonstrated that recombinant, nonglycosylated FcγRIIIa produced in *E. coli* exhibits no significant conformational differences compared with the glycosylated wild-type receptor expressed in HEK cells. Furthermore, crystal structures of the FcγRIIIa–IgG complex show that although the terminal galactose residue of the IgG Asn297 N-glycan does not directly interact with the modified nonglycosylated FcγRIIIa ligand,^27^ the overall IgG–receptor interaction remains unaffected.

As a further proof of this, in one recent study, we demonstrated that the TSKgel FcR-IIIA-NPR affinity column can separate fucosylated and afucosylated glyco-variants of rituximab. More specifically, when analyzing two rituximab glyco-variants, the G0F variant exhibited shorter retention on the column compared to the afucosylated G0 variant, with a clear and measurable difference in retention time.^28^

These experimental findings clearly support the use of commercial FcγRIIIa affinity columns based on recombinant nonglycosylated receptor to successfully separate IgG glycoforms based on their relative receptor-binding affinities. Studies have shown that this analytical method can be used to evaluate the functional glycosylation profile of mAbs, for instance, confirming the positive effect attributed to high degree of terminal galactosylation in recombinant therapeutic mAbs.^29^

While this technique has proven valuable for assessing recombinant anticancer antibodies, its application to human antibodies produced in response to viral infections remains largely unexplored. These endogenous antibodies are produced from B cells and often exhibit a different glycan composition compared to recombinant counterparts.^19^ Previous studies also demonstrated that antibodies generated in response to either SARS-CoV-2 natural infection or vaccination can mediate NK cell-driven ADCC. However, antibodies elicited by natural infection have been shown to possess a significantly greater ability to induce ADCC compared with those produced following vaccination.^30^ Despite these observations, earlier reports have provided limited insight into the structural and glycosylation features of mAbs that underline these functional differences, and the role of glycan composition in modulating ADCC remains poorly characterized, warranting further investigation.

The present work provides novel insights by directly evaluating how naturally occurring human glycoform variants influence Fc-mediated effector function, specifically ADCC activity. While the role of Fc glycan structures—particularly terminal galactose and sialic acid—in enhancing ADCC is well established in the context of glycoengineered anticancer monoclonal antibodies, this relationship has not been systematically investigated for human monoclonal antibodies naturally produced during viral infections (anti-SARS-CoV-2) in convalescent or vaccinated individuals.

In this study, we characterize distinct glycoform profiles of antibodies isolated from individuals exposed to SARS-CoV-2 variants and correlate these naturally occurring differences—particularly the levels of terminal galactose and sialylation—with their ability to activate NK cells. Specific anti-SARS-CoV-2 IgG1 human monoclonal antibodies (hmAbs1–3) from B-cell clones have originated from the polyclonal antibody response elicited by SARS-CoV-2 vaccination (hmAbs1–2) or infection (hmAbs3).

These hmAbs have been analyzed using a three-pronged approach: (i) stepwise elucidation of glycosylation profiles of the previously uncharacterized hmAbs using advanced analytical techniques in combination with FcγRIIIa affinity-based analyses; (ii) isolation of the hmAb glycoforms by FcγRIIIa affinity chromatography; and (iii) functional evaluation of hmAb-mediated ADCC activity by an optimized assay.

This approach allowed us to identify the hmAb derived from an infected subject (hmAbs3) as the most efficient SARS-CoV-2-specific hmAb, characterized by the best efficiency in contrasting SARS-CoV-2 infection, in terms of both ADCC and neutralizing activity.

This study highlights a novel integrative workflow that bridges glycan structural analysis with functional assessment, aiming to develop hmAbs that combine optimal neutralizing and ADCC activities and display enhanced therapeutic potential against SARS-CoV-2 or other viral infections.

The proposed multidisciplinary function-oriented characterization strategy enabled the identification of functionally active glycoforms, facilitating the selection of optimal hmAb candidates for therapeutic applications, which might include the isolation of selected glycovariants, as well as the design of suitable glycoengineering strategies to obtain optimal ADCC activity in the case of recombinant hmAbs.

## 2 Results and Discussion

hmAbs targeting the SARS-CoV-2 RBD of the Spike protein were obtained from B cells isolated from SARS-CoV-2-vaccinated (hmAb1 and hmAb2) individuals or from one SARS-CoV-2 convalescent (hmAb3) subject (Figure S1). Their neutralizing activity toward ancestral virus and its subsequent variants of concern was assessed in the Vero-E6 cell-based system.

Given the therapeutic relevance of the mAbs for the treatment of SARS-CoV-2 and its variants, a systematic workflow was implemented to comprehensively characterize their proteoforms and glycosylation composition as a preliminary requirement for a specific glycan-activity relationship study. The study initially began with intact mass analysis to determine the overall molecular weight of Abs, providing a first assessment of their heterogeneity. This was followed by subunit analysis, focusing on the Fc region to resolve and identify specific glycoforms. Subsequently, released N-glycan analysis was performed to profile the complete glycan composition of each hmAb sample. To assess functional relevance, FcγRIIIa affinity chromatography was employed to separate glycoforms based on their binding affinities to the receptor, thereby enriching functionally distinct populations. Finally, a surrogate ADCC bioassay was conducted to evaluate the therapeutic potential of the individual glycoform fractions isolated through FcγR affinity separation. This approach aims at the selection of hmAb candidates possessing the highest neutralizing and ADCC activities for antiviral therapeutic application.

### 2.1 Evaluation of the Neutralizing Activity

The SARS-CoV-2 infection is mediated by the binding between its RBD protein and the ACE2 receptor expressed on target cells. The Abs’ ability to target the virus Spike protein in the RBD-binding site provides the neutralizing effect, resulting in infection inhibition that can be harnessed in an immunotherapeutic approach to counteract the disease.

With the aim to select optimal hmAbs, we evaluated the neutralizing activity of hmAbs1, 2, and 3 toward ancestral SARS-CoV-2, the D614G and subsequent VoCs Beta, Delta, and Omicron (XBB.1.5 and EG.5.1). As shown in Figure 2A,B, hmAb1 did not neutralize the SARS-CoV-2 VoCs. In contrast, hmAb2 neutralized the Wuhan, D614G, and Delta variants, with low activity also against Beta. Interestingly, hmAb3 showed both the strongest neutralization profile, being active at nanoconcentrations, and the broadest cross-reactivity by blocking also Beta and the more recent Omicron EG.5.1 variant. None of the hmAbs showed neutralizing activity against the Omicron XBB.1.5 variant.

**Figure 2:**
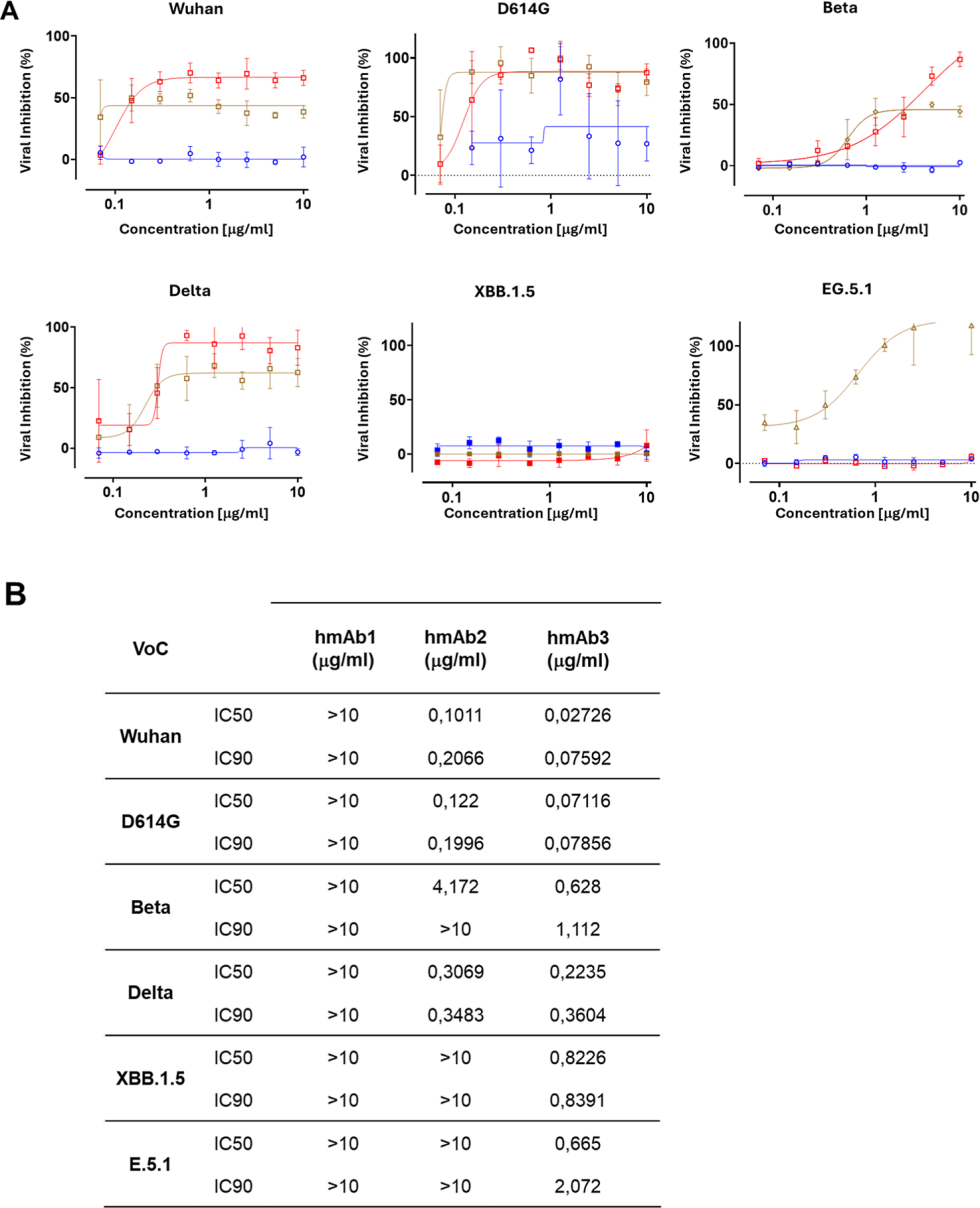
Neutralizing activity of hmAbs1 (Blue), 2 (Red), and 3 (Brown) against SARS-CoV-2 VoCs. (A) Nonlinear regression of hmAb activity, expressed as the percentage of viral inhibition. (B) Inhibitory concentrations of hmAbs required to have a half-maximal (IC50) or total (IC90) neutralization of SARS-CoV-2 VoCs.

### 2.2 Intact Mass Analysis of Purified Anti-SARS-CoV-2 Human Monoclonal Antibodies

Intact mass analysis was performed on purified antibody samples using RP-HRMS. Deconvoluted mass spectra revealed that the predominant proteoforms of hmAb1, hmAb2, and hmAb3 exhibited molecular masses of ∼149 607 Da, ∼149 184 Da, and ∼148 303 Da, respectively. These results aligned with the expected molecular weight of IgG subclasses excluding IgG3 (∼146 kDa) while reflecting variations due to post-translational modifications (PTMs), primarily glycosylation.^31^ Each hmAb sample exhibited a broad distribution of proteoforms under a single chromatographic peak in the reverse-phase liquid chromatography (RP-LC) profile (Figure S2A). To specifically assess glycosylation, the antibodies were treated with PNGase F to enzymatically remove N-linked glycans. Subsequent RP-HRMS analysis of the deglycosylated samples showed deconvoluted molecular masses of ∼146 355 Da (hmAb1), ∼145 293 Da (hmAb2), and ∼144 637 Da (hmAb3) (Figure S2B). Although enzymatic deglycosylation reduced the overall mass and confirmed the presence of N-glycans, the broad proteoform distribution observed even after PNGase F treatment limited precise glycoform characterization. Interestingly, hmAb1 and hmAb2 exhibited ∼62 Da molecular mass difference after deglycosylation, implying that the initial differences in their intact masses were largely attributable to variations in glycan composition. This suggests that while their protein backbones were similar, they likely possessed distinct glycosylation profiles. In contrast, hmAb3 showed a significantly lower deglycosylated mass, indicating structural differences at the polypeptide level or the presence of additional modifications, which could reflect a different expression history or possibly an immune response to a distinct SARS-CoV-2 variant.

### 2.3 Subunit Mass Analysis

Partial digestion of hmAb samples using the IdeS enzyme, followed by reduction with dithiothreitol (DTT), generated distinct subunits of approximately 25 kDa, namely, Fd′, light chain (LC), and scFc. IdeS specifically cleaves the mAbs below the hinge region, yielding scFc and F­(ab′)_2_ fragments, which, upon reduction, further dissociate into their respective subunits.^32^ The fragments of all three hmAb samples were analyzed using hydrophilic interaction liquid chromatography–mass spectrometry (HILIC-MS) to investigate the glycoform distribution. The Fd′ and LC subunits, which lack glycosylation, exhibited lower retention on the HILIC column and eluted earlier. In contrast, the scFc subunits, containing N-linked glycans, demonstrated increased retention and resolved into multiple peaks, corresponding to different glycoforms (Figure S3). In the absence of full sequence information for the hmAbs, the assignment of Fc glycoforms was performed by matching the observed subunit masses to the corresponding theoretical Fc/2 masses reported in the literature for conserved IgG1 Fc sequences.^33^ Specific mass differences corresponding to common glycoforms (e.g., G0, G0F, G1F, and G2F) were used for identification. Deconvolution of mass spectra using UniDec software provided precise molecular masses for each subunit, allowing for the identification and relative quantification of glycoforms. Overall, the hmAb samples exhibited glycosylation patterns characteristic of human IgG antibodies (summarized in Table 1). The predominant N-glycan structures included corefucosylated biantennary glycans with varying degrees of galactosylation. Specifically, G0F accounted for approximately 25% of the total glycan population, while G1F and G2F species were present at roughly 20% each. In addition to these major species, the hmAbs also displayed a notable proportion (10–20%) of more structurally complex glycoforms, including bisected glycans (containing a bisected N-acetylglucosamine) and sialylated variants (bearing one or more sialic acid residues). These glycoforms are commonly observed in endogenous IgG and reflect typical human immune glycosylation profiles.^19^

**Table 1:** Deconvoluted Molecular Mass and Relative Abundance of Fragments of All Three hmAb Samples Analyzed by HILIC-MS after the Treatment of IdeS and DTT

subunit	hmAb1	hmAb2	hmAb3	
Fd	25677	25400	25065	
LC	23605	23346	23482	

scFc	Observed MW (Da)	hmAb1	hmAb2	hmAb3
		relative abundance (%)		
G0	25087	ND^a^	ND^a^	1.89
G0 + K	25198	0.61	ND^a^	ND^a^
G0F	25232	11.23	0.53	3.98
G0F + K	25360	16.79	3.18	2.68
G0F + B + K	25563	7.70	ND^a^	ND^a^
G0F + B	25434	ND^a^	1.16	ND^a^
G1	25248	ND^a^	3.05	10.19
G1F	25394	4.19	18.06	17.55
G1F + K	25522	19.62	14.94	7.94
G1F + B + K	25725	18.98	15.00	4.46
G1F + B	25597	2.03	14.87	11.33
G2	25407	ND^a^	ND^a^	6.99
G2F	25556	10.81	10.62	13.89
G2F + K	25684	3.70	12.75	6.01
G2FS1	25847	1.23	3.18	2.56
G2FS1 + K	25975	1.23	2.12	8.22
G2S2	25993	ND^a^	ND^a^	0.64
G2FS1 + Hex + K	26138	0.61	ND^a^	ND^a^
G2FS1 + Hex	26012	ND^a^	0.53	ND^a^
G2FS1 + B + K	26178	ND^a^	ND^a^	0.50
G2FS1 + B	26051	ND^a^	ND^a^	1.09

a ND: Not detectable, Hex: Hexose, B: Bisected N-acetylglucosamine, K: lysine.

The relative abundances of these glycoforms are presented in Figure 3. Subunit analysis of hmAb1 samples revealed a diverse array of glycoforms within the Fc fragment, reflecting distinct glycosylation patterns that contribute to overall glycan heterogeneity. The afucosylated glycoform exhibited a molecular mass of approximately 25 198 Da. Mass shifts associated with glycosylation variations were observed, including an increase of ∼162 Da corresponding to the addition of a single galactose residue and a shift of ∼145 Da indicative of core fucosylation.^34^ Additionally, a mass reduction of ∼128 Da was attributed to C-terminal lysine clipping, a well-documented PTM in mAbs.^35^ Human serum-derived mAbs are characterized by a higher degree of glycan heterogeneity compared to recombinant therapeutic recombinant mAbs.^19^ In line with the literature, the hmAb samples obtained from a vaccinated (hmAb1 and 2) or from a COVID-19-recovered (hmAb3) individual exhibited markedly diverse and complex glycosylation profiles. The predominant glycoform in hmAb1 was G0F, accounting for ∼28% of the total glycoforms identified. This was followed by G1F (∼23.8%), bisected GlcNAc-containing G1F + B (∼19%), and G2F (∼14.51%). Terminal sialic acid residues, which add structural complexity to glycans, were identified in low-abundance glycoform G2FS1 (∼2.5%). In contrast to hmAb1, a different glycosylation profile was observed in hmAb2 samples, where the most abundant was the bisected GlcNAc-containing G1F + B which contributed ∼29% of the overall glycoform identified, followed by G1F (∼23%) and G2F (∼23%). Interestingly, G0F was present in lower amounts (∼4%) compared with the hmAb1 sample. The subunit analysis of hmAb3 revealed that G1F was the most prevalent, accounting for 25.49% of the total, followed by G1F–B at 15.79%. G2F was identified as the second most abundant, comprising 20% of the total glycoform population. Notably, the sialylated G2FS1 was present in a higher proportion (10%) compared with hmAb1 and hmAb2 samples. Additionally, G2, G2S2, and G2FS1-B were detected only in hmAb3 contributing ∼10% of total glycoforms identified, whereas they were absent in the other hmAb samples.

**Figure 3:**
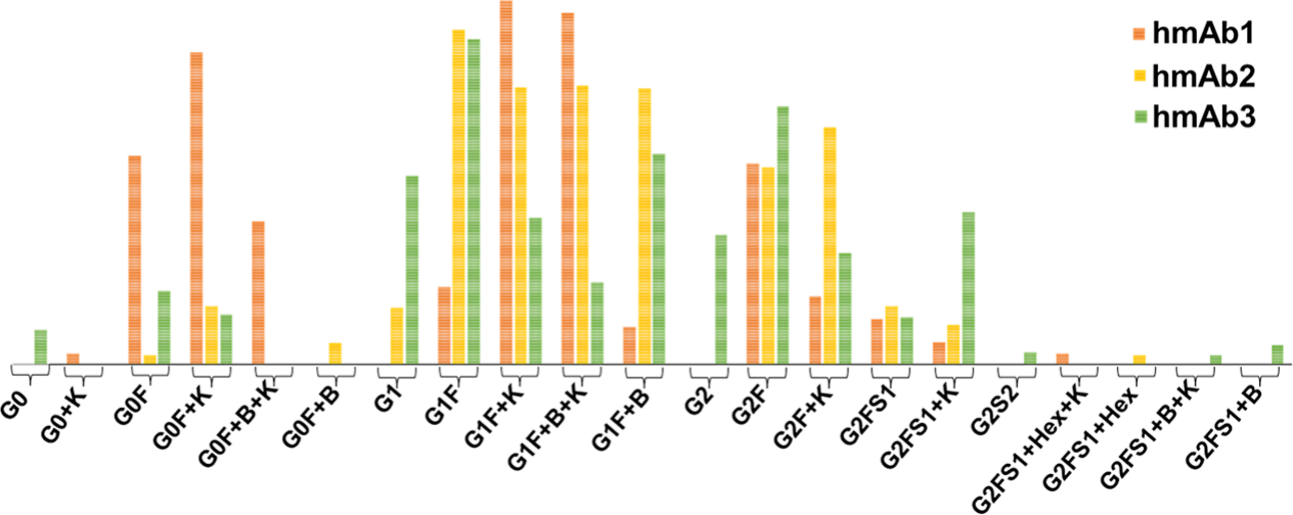
Distribution and relative abundance of glycoforms identified in the three hmAb samples.

### 2.4 Released N-Glycan Analysis

Released N-glycan analysis is a widely utilized method for the relative quantification of glycans in mAb products. After enzymatic deglycosylation of the mAb using PNGase F, the released N-glycans are commonly derivatized at their reducing ends with fluorescent tags to enhance stability and improve ionization efficiency for mass spectrometric analysis.^36^ In this study, the released N-glycan profiling of the three hmAb samples was performed using procainamide labeling, followed by mass spectrometric analysis to assess glycan composition and relative abundances. The relative abundance of N-glycan identified is presented in Figure 4. The results revealed distinct glycosylation patterns across the hmAb samples, highlighting variations in core fucosylation, bisected GlcNAc, high-mannose structures, and sialylation (Table S1). Among the detected glycans, G0F (*m*/*z* = 1682.73) was one of the most abundant across all samples, with the highest relative abundance observed in hmAb1 (33.97%), followed by hmAb2 (13.54%), whereas hmAb3 contained only 6.11%. Other fucosylated glycans, such as G1F (*m*/*z* 1844.78) and G2F (*m*/*z* 2006.83), were present in all hmAb samples, but hmAb3 and hmAb2 exhibited a higher proportion of G2F (13.41% and 12.56%, respectively) compared to hmAb1 (7.99%), indicating increased galactosylation in these samples.

**Figure 4:**
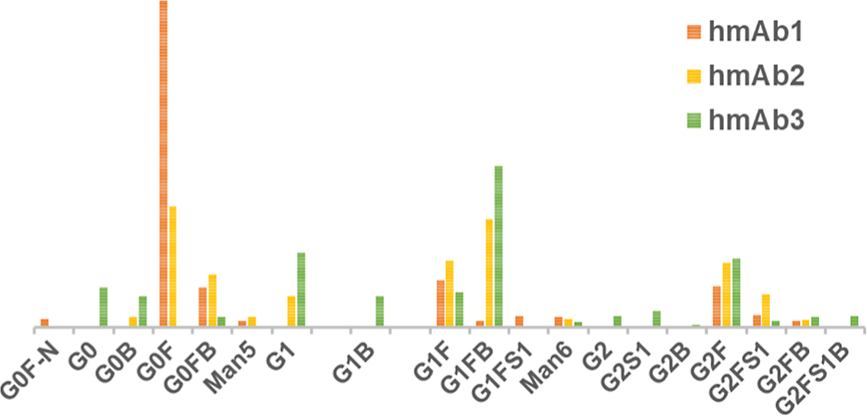
Distribution and relative abundance of released N-glycan identified in the three hmAb samples.

Bisected glycans also showed sample-specific variations. G0B (*m*/*z* 1739.75), a bisected nonfucosylated glycan, was only detected in hmAb2 (1.89%) and hmAb3 (6.11%), whereas it was absent in hmAb1. Similarly, G1FB (*m*/*z* 2047.86), a fucosylated and bisected glycan with one Gal unit, was found at a significantly higher level in hmAb2 and hmAb3 (around 21%), while hmAb1 exhibited a much lower abundance (11.11%). High-mannose glycans, such as Man5 (*m*/*z* 1454.62) and Man6 (*m*/*z* 1616.67), were detected at relatively low levels across all samples (<2.5%). Sialylated glycans were also observed in all the samples: G1FS1 (*m*/*z* 2135.88), a monosialylated glycan, was only detected in hmAb1 (2.11%), while G2FS1 (*m*/*z* 2298.93) was found in all samples, with the highest abundance in hmAb2 (6.39%).

Previous studies have shown that glycosylation on the FcγR receptor enhances its interaction with glycosylated mAbs.^37^ Lippold et al. developed an FcγR-coupled mass spectrometry approach to analyze IgG–FcγR interactions in a proteoform-resolved manner, demonstrating the method on a panel of nine monoclonal antibodies, including nonbinding, low-affinity, and high-affinity Fc-engineered or glycoengineered mAbs.^38^ FcγRIIIa affinity chromatography was adopted here to dissect and systematically assign Fc glycoforms to their functional attributes and in particular to isolate low-heterogeneous populations of glycoforms of IgGs to be used in cellular assays.

Overall, both subunit and released N-glycan analyses of the hmAb samples revealed distinct and antibody-specific glycosylation profiles. In hmAb1, the glycan composition was dominated by G0F and G1F structures, which together comprised approximately 50–60% of the total glycan pool. In hmAb2, the predominant glycoforms included G1F, G2F, and the bisected variant G1FB, collectively accounting for around 60% of the glycan content, while Neu5Ac-α2,6-Gal forms resulted at around 4.5%. In contrast, hmAb3 exhibited a more heterogeneous and evenly distributed glycan profile. The major glycoforms G1F, G2F, bisected G1FB, and sialylated species were present at comparable levels, together representing approximately 70–80% of the total glycan content (with sialylated sugars accounting for more than 8%). This broader and more balanced glycosylation pattern suggests increased structural complexity in hmAb3, potentially reflecting variations in the immune response.

### 2.5 FcyRIIIa-Based Affinity Chromatography Separation of Glycoforms

To further elucidate the functional implications of these glycan variants, a targeted analytical approach combining FcγRIIIa-based affinity chromatography with high-resolution mass spectrometry was employed. This integrative method enabled the selective enrichment and characterization of functionally relevant glycoforms, allowing for a deeper understanding of their contribution to Fc-mediated effector functions.

FcγRIIIa exhibits a moderate affinity for the Fc region of mAbs. The receptor’s affinity is primarily influenced by the composition of N-glycans within the heavy chains.^39^ The mAb sample was loaded onto a commercial FcγRIIIa affinity column under near-neutral pH buffer conditions to facilitate antibody–receptor interactions. Separation of glycoforms is achieved by gradually lowering the pH, thereby disrupting the interaction. Numerous studies have evaluated different mobile phase compositions, ranging from nonvolatile to MS-compatible volatile mobile phases. Notably, a linear pH gradient in the mobile phase is considered optimal for such analyses.^29,40^ In this study, affinity chromatography was directly coupled to MS using a mobile phase containing 50 mM ammonium acetate. The hmAb samples (1–3) were resolved into five distinct peaks, each with varying receptor affinities and abundances (Figure 5). Glycoforms with lower affinity for FcγRIIIa eluted first, followed sequentially by glycoforms with moderate to high affinity.

**Figure 5:**
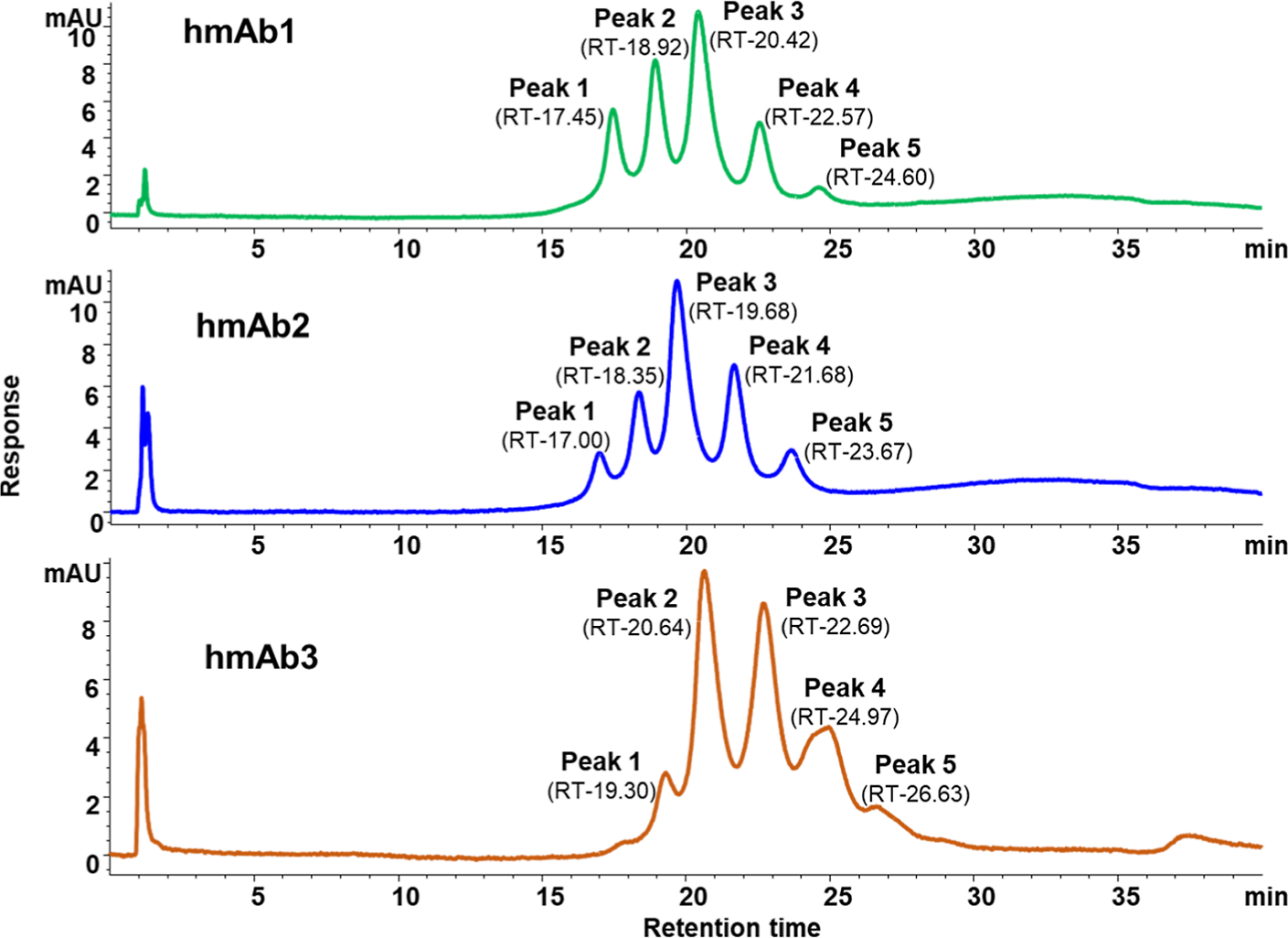
FcγRIIIa affinity chromatography profiles of the hmAb samples were analyzed using a UV detector at an absorbance wavelength of 280 nm.

The final peak corresponded to glycoform with the highest receptor-binding affinity. Analysis revealed an *m*/*z* range of 5000–7000, with charge states ranging from +21 to +30 across all glycoform peaks. Furthermore, to further characterize the glycoforms, each eluted fraction from the FcγRIIIa affinity column was collected and subjected to enzymatic deglycosylation using PNGase F, which removes N-glycans from the Fc region. The deglycosylated fractions were then analyzed using RPLC-MS to measure the intact mass (Figure S4). Comparing the intact masses of glycosylated and deglycosylated fractions enabled the precise assignment of glycoforms to each FcγRIIIa-bound peak (Table 2). The integration of intact mass analysis with FcγRIIIa affinity chromatography and subsequent enzymatic deglycosylation offers a systematic and robust strategy for the functional characterization of glycoforms in uncharacterized hmAbs.

**Table 2:**
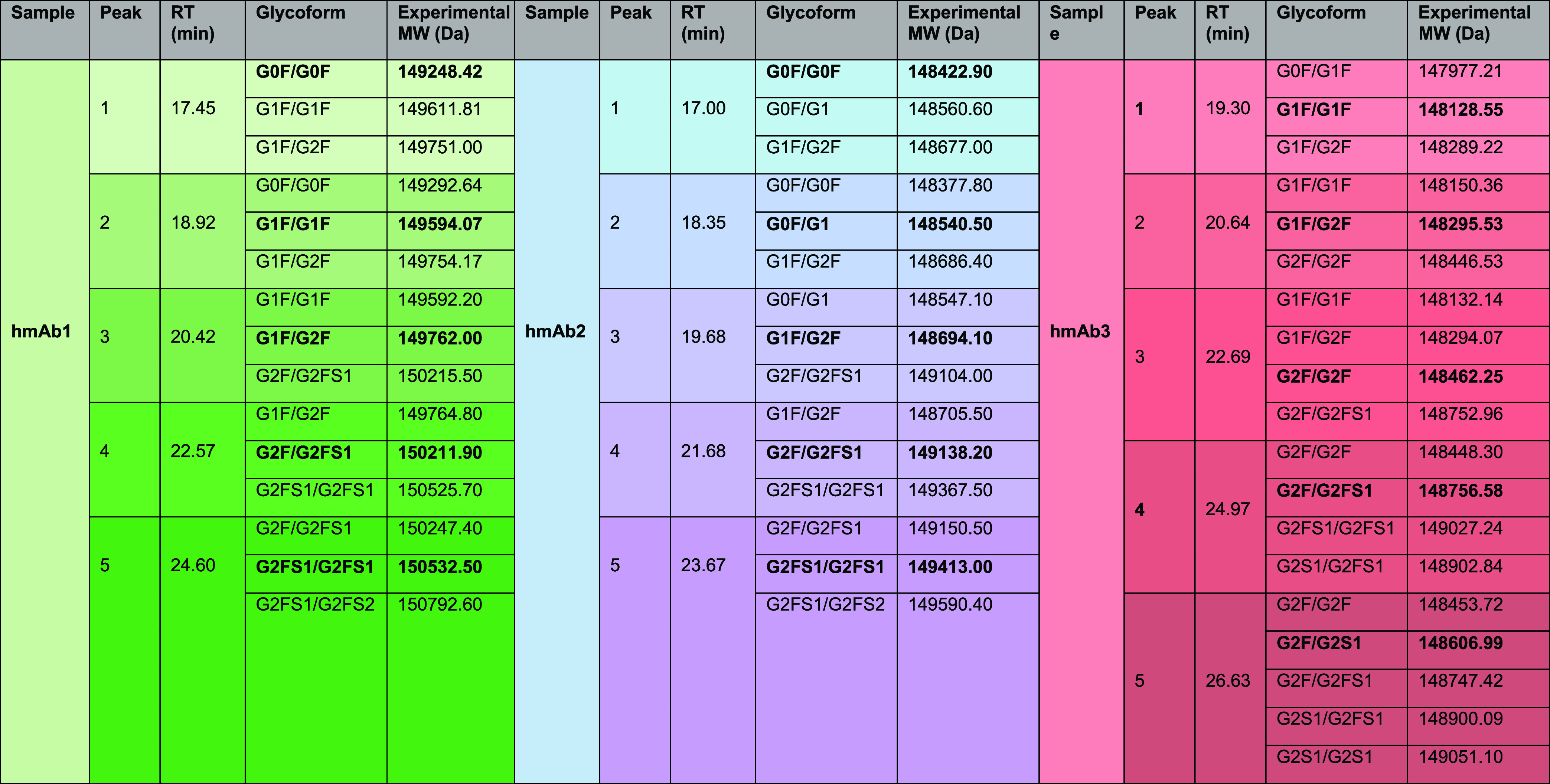
Glycoform Assignment of hmAb1, hmAb2, and hmAb3 Monoclonal Antibody Samples by Intact Mass Analysis of the Individual Eluting Peaks in the FcγRIIIa Affinity Column

The glycosylation analysis of the hmAb1 sample revealed that the peak rich in G1F/G2F (Peak 3) exhibited the highest relative abundance at 37.72%, making it the predominant glycoform in the sample.

This was followed by the peak rich in G1F/G1F (Peak 2, 23.12%), G0F/G0F (Peak 1, 18.55%), and G2F/G2FS1 (Peak 4, 15.36%). G2FS1/G2FS1 (Peak 5) was the least abundant, contributing only 5.31% of the overall glycoform profile (Figure 6A). These glycoform compositions highlight the structural diversity of the hmAbs. Peak 1, predominantly composed of the G0F/G0F, exhibited the lowest affinity for the FcγRIIIa receptor.^41^ Conversely, Peaks 2 and 3, which are enriched in G1F/G1F and G1F/G2F, showed intermediate to high receptor affinity. The increased terminal galactosylation in these glycoforms enhances FcγRIIIa binding. Notably, the enhanced receptor affinity observed for glycoforms with terminal galactose agrees with earlier reports indicating that galactosylation significantly impacts Fcγ receptor interactions and biological activity^39^ and confirm that the immobilized mutant commercial receptor maintains a similar selectivity as reported for WT-glycosylated human FcγRIIIa receptor. Peak 5—characterized by the G2FS1/G2FS1 glycoform—exhibited the strongest receptor binding in our analysis. This could be due to the presence of terminal sialic acid residues, which increase glycan complexity and are known to enhance receptor interactions and anti-inflammatory properties.^42^ Recent studies have shown that increased glycan complexity on mAbs not only enhances their retention on the FcγRIIIa affinity column^17^ but also correlates with stronger binding affinity, as confirmed by SPR analysis.^23^ Specifically, mAbs bearing the Neu5Ac-α2,6-Gal motif demonstrated significantly higher affinity for the FcγRIIIa receptor. Additionally, sialylation has been shown to extend serum half-life and modulate mAb effector function.^21^

**Figure 6:**
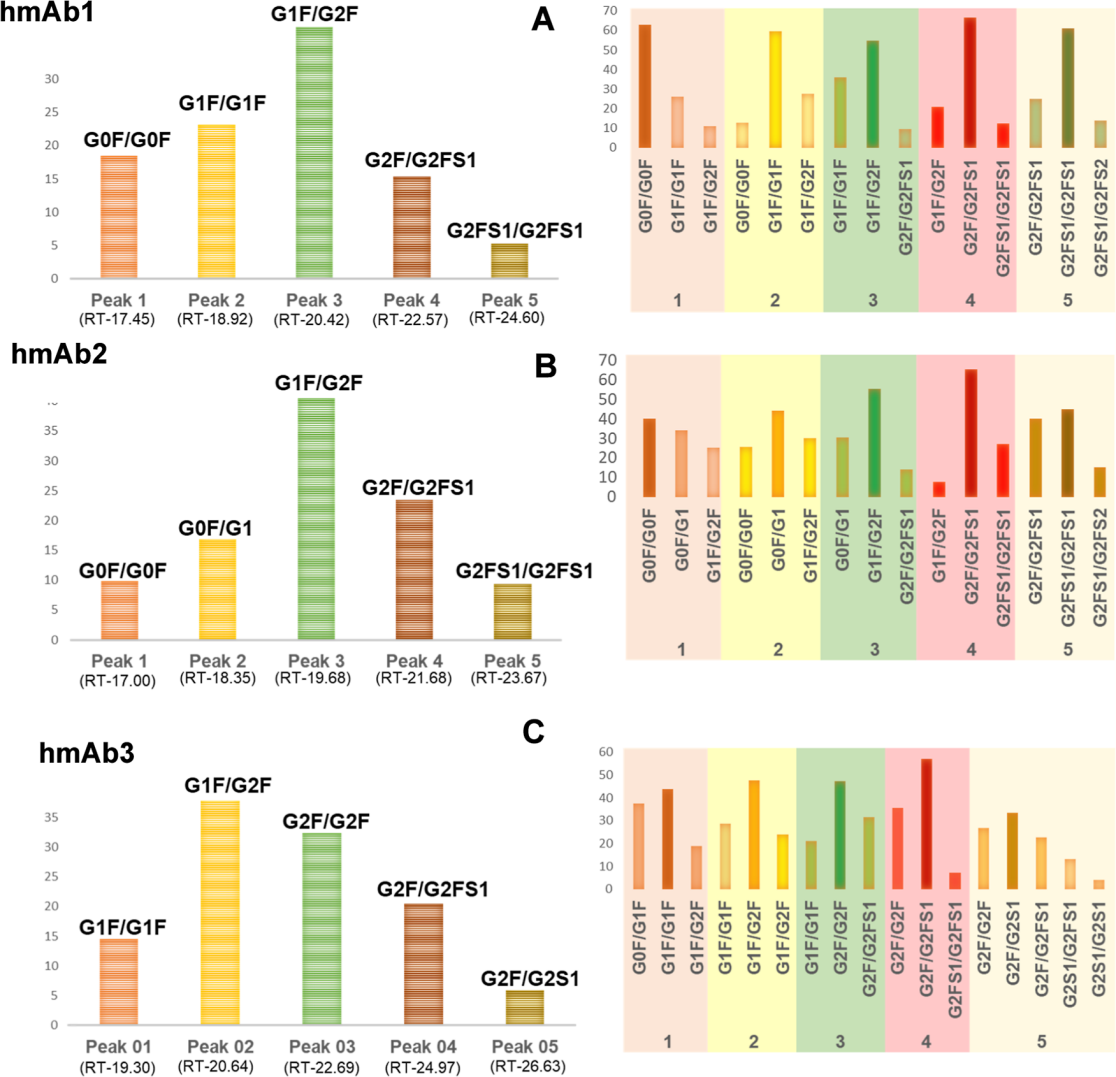
hmAbs (hmAb1, hmAb2, and hmAb3) were analyzed using FcγRIIIa affinity-mass spectrometry (A–C). The relative abundance of most abundant glycoforms for each peak is shown, along with the specific glycoform distributions corresponding to each peak.

For the hmAb2 sample, the affinity chromatography profile closely resembled that of the hmAb1 sample; however, the glycoform composition exhibited some differences because of the presence of a non-negligible quantity of afucosylated species in peaks 1–3 (Figure 6B).

Indeed, peak 2 predominantly displayed an afucosylated G1 on one arm of the heavy chain, in higher quantities compared with peak 1, leading to increased retention on the FcγRIIIa affinity column. In fact, afucosylation is well-documented to significantly increase the binding affinity of IgG Fc regions to FcγRIIIa.^43^

The analysis of the hmAb3 sample demonstrated a peculiar glycoform profile with a notable enrichment of high-terminal galactose-containing structures, which are known to positively influence FcγRIIIa binding and effector function (Figure 6C). Peak 2 and Peak 3 are composed of a high quantity of G1F/G2F (predominant in peak 2 with G1F/G1F present in a similar amount) and G2F/G2F (predominant in peak 3), each peak representing approximately 30% of the total sample. These glycoforms are characterized by a high galactose content, which has previously been reported to enhance receptor affinity by prolonging retention on affinity columns.^26^ Peak 4 and Peak 5 displayed unique glycoforms compared to hmAb1 and hmAb2 samples because they contain afucosylated/sialylated glycans.

Notably, G2F/G2S1 is the predominant glycan in peak 5 that also contains G2S1/G2FS1 and small amounts of G2S1/G2S1. The presence of terminal sialic acid residues in this glycoform (in addition to the absence of fucose) significantly increased their retention on the FcγRIIIa affinity column due to enhanced receptor interactions. Peak 4 accounted for 21.84% of the total glycoform abundance, while Peak 5 approximately contributed 8%. These glycoforms, with their distinctive sialylation patterns, underline the structural complexity and functional diversity of hmAb3, distinguishing it from hmAb1 and hmAb2. In contrast, Peak 1 contained G1F/G1F, representing approximately 8% of the total glycoform distribution, with lower galactose and sialic acid contents contributing to reduced receptor affinity. The enrichment of terminal galactose and sialic acid residues in specific glycoforms observed in hmAb3 further supports the trends identified in hmAb1 and hmAb2, emphasizing the critical role of terminal glycosylation in modulating FcγRIIIa binding and the overall therapeutic efficacy. This is in agreement with prior studies demonstrating the impact of glycan composition on mAb function, including enhanced receptor binding, improved pharmacokinetics, and altered effector function.^42,44^

Overall, the three hmAbs exhibited distinct glycoform compositions and varying interactions with FcγRIIIa, as observed through affinity chromatography. In the case of hmAb1, approximately 60% of the total antibody population interacting with FcγRIIIa consisted of G1F/G2F and G1F/G1F. For hmAb2, the major interacting glycoforms were G1F/G2F and G2F/G2FS1, together accounting for 63% of the FcγRIIIa-bound fraction. Similarly, hmAb3 showed a 61% contribution from G1F/G2F and G2F/G2F, along with an additional 15% contribution from the sialylated G2FS1 variant.

### 2.6 Fc Glycoforms Impact on ADCC

To measure the Fc-mediated function of mAbs, several assays have been optimized during the SARS-CoV-2 pandemic.^45^ For instance, the fluorescence-reduction assay measured the killing by determining the decrease of intensity of a fluorescent molecule contained in Spike + target cells.^46^ In the CD16 reporter assay, the effector activity was instead evaluated as an increased luciferase signal in the CD16 expressing cell line after incubation with transfected Spike + target cells and plasma or Abs. Another way to assess the effector cell activation was to observe the upregulation of cell-surface markers, as well as the production of cytotoxic molecules in the presence of infected or transfected target cells. In this study, we optimized a surrogate ADCC assay performed in the absence of target cells. This approach allowed for the measurement of the effector cell degranulation directly due to antibody-mediated Fc receptor engagement. To this end, peripheral blood mononuclear cells (PBMC) were plated without or in the presence of coated hmAbs, and the NK cell degranulation was assessed by measuring their surface expression of CD107a as a proxy for cytotoxic activity. The donor-dependent variability in FcγRIIIa glycosylation, depending on PTMs or polymorphisms, could influence the interaction with Fc-CD16a,^47^ thereby introducing an additional layer of complexity into the assessment of effector function. Based on these premises and considering that our aim was to establish a controlled experimental model, we conducted our studies using PBMCs derived from a single healthy donor. Although we did not characterize the donor FcγRIIIa glycosylation, the ADCC readouts showed consistent relative differences between enriched glycoform fractions and correlated with the glycosylation profile.

As shown in the gating strategy plots (Figure 7A,B), degranulating NK were identified among lymphocytes as CD3neg/CD56pos, CD107a expressing cells. We tested three different concentrations of hmAb1–3 (10, 20, and 40 μg/mL), and as shown in Figure 7C, hmAb3 induced the highest NK cell activation by prompting the greater percentage of CD107a-positive NK cells at all concentrations used. This enhanced activity correlated with affinity chromatography results, which indicated that hmAb3 had a higher proportion of G2F/G2F and G2F/G2FS1 glycoforms compared with the other hmAb samples. Based on these observations, hmAb3 was selected for subsequent experiments. To evaluate whether distinct Fc glycoforms of hmAb3 influenced NK cell-mediated cytotoxicity, five chromatographically resolved fractions (F1–F5, corresponding to peaks 1–5) from the FcγRIIIa affinity profile of the hmAb3 sample were collected and subjected to the surrogate ADCC assay, at a concentration of 10 μg/mL each. However, under these conditions, no differences were observed between the whole hmAb3 and its isolated fractions corresponding to specific glycoforms (Figure S5), suggesting that saturating concentrations were used. Hence, we repeated the assay using 5 μg/mL hmAb3 and its fractions. In these conditions, F3 induced a significant increase in ADCC compared with the whole hmAb3 and F1 (Figure 7D). This activity could be due to its high abundance in Galactose (G2). Also, F5 displayed strong activity, inducing a significantly higher NK cell degranulation than hmAb3 and F1 (Figure 7D). Detailed glycan profiling of F5 revealed a predominance of complex, highly galactosylated and sialylated structures, notably G2F, G2FS1, and G2S1. Previous studies have demonstrated that the presence of the terminal Neu5Ac-α2,6-Gal motif is associated with increased conformational flexibility of the Fc region, thereby enhancing its interaction with the FcγRIIIa receptor and boosting ADCC activity.^23^ Our findings are consistent with these observations. The enhanced NK cell activation and strong receptor binding exhibited by F5 suggest that terminal sialylation plays a critical role in modulating the effector function of Abs. This could be harnessed to generate glycoengineered human B-cell-derived therapeutic mAbs with a boosted ADCC activity.

**Figure 7:**
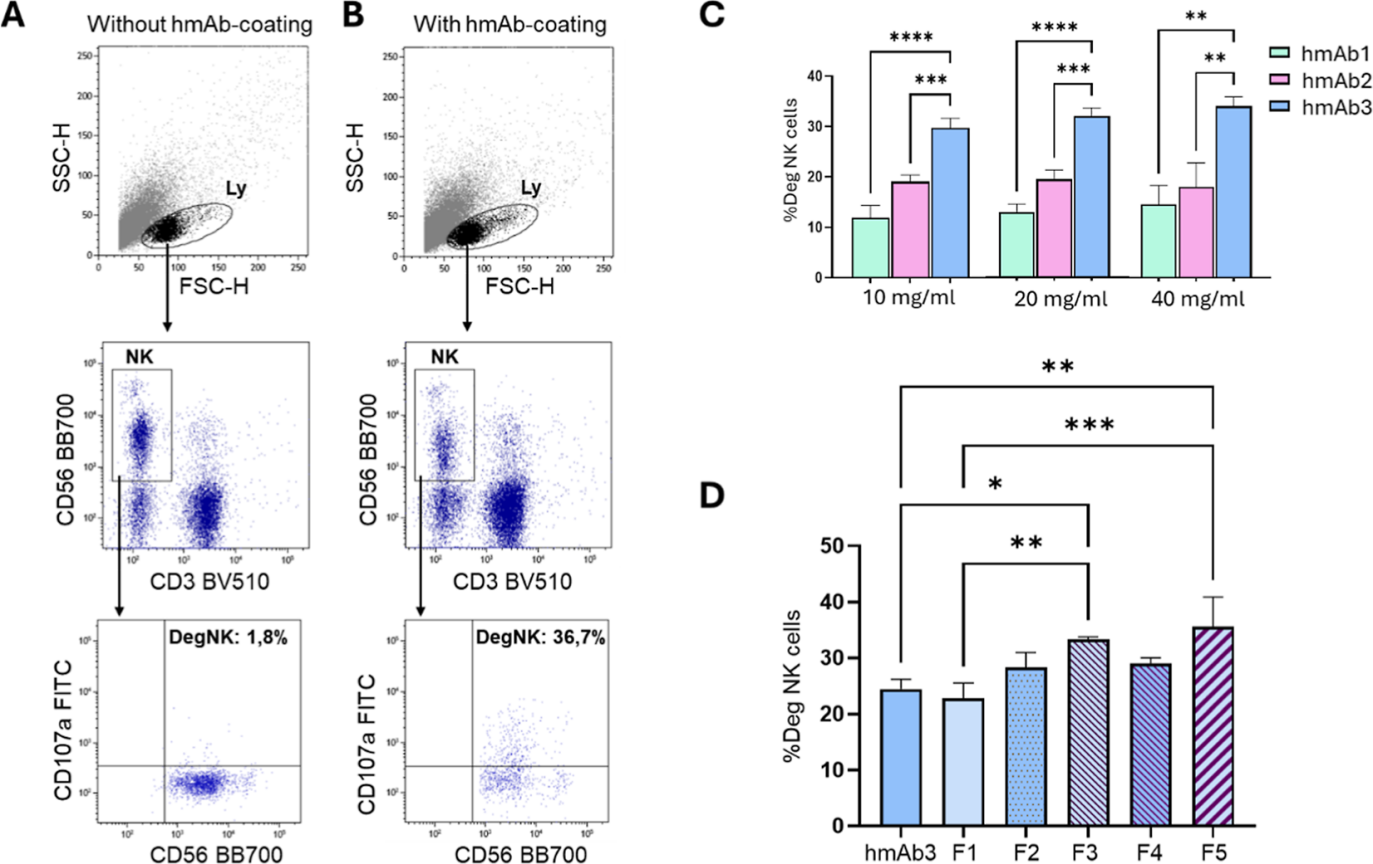
hmAb cytotoxic activity was measured by flow cytometry. (A,B) Gating strategy to quantify degranulating NK (DegNK) cells among lymphocytes (Ly). Degranulating activity of NK cells mediated by several concentrations of hmAb1–3 (C) or by 5 μg/mL of the hmAb3-resolved fractions (F1–5, (D)). In (C,D), the percentage of DegNK without hmAb-coating was subtracted from %DegNK with hmAb-coating. The one-way ANOVA test was used. ****p < 0.0001, ***p < 0.001, **p < 0.01, and *p < 0.05. Experiments were performed in triplicate.

The fractionation and glycan analysis performed in this study not only underscore the extensive glycan heterogeneity among human-derived mAbs but also provide functional insights into how specific glycan features influence immune effector responses.

The analysis of the glycoforms contained in hmAbs highlights the potential functional advantages of this product in comparison to those of others. These findings contribute to a deeper understanding of how glycosylation patterns (high galactosylation and sialylation degrees) influence FcγRIIIa binding and potentially affect therapeutic efficacy.

In addition, our results offer compelling evidence that mAbs obtained from B cells of convalescent individuals exhibit more favorable glycan profiles—characterized by higher levels of galactosylation and sialylation—compared with those derived from vaccinated individuals. These structural distinctions likely contribute to the superior ADCC activity and therapeutic potential observed in antibodies elicited by a natural SARS-CoV-2 infection.

## 3 Conclusions

HmAbs exhibit complex glycosylation patterns that can significantly influence their functional activity, particularly Fc-mediated effector functions such as ADCC. Thus, to develop efficient hmAbs for antiviral therapeutic application, in addition to the evaluation of virus neutralizing activity, characterization of the optimal glycosylation profile for ADCC function seems mandatory. This study demonstrates the feasibility and efficacy of the proposed workflow used for the characterization of the glycosylation profiles of three IgG1 hmAbs targeting the Spike protein of SARS-CoV-2 and generated from B cells isolated from either vaccinated (hmAb1 and hmAb2) or COVID-19 convalescent subject (hmAb3). The hmAb3 resulted with the highest neutralizing and ADCC activity, with the latter that seems correlated with a higher content, compared with the other hmAbs, of glycans with a terminal galactose or Neu5Ac-α2,6-Gal epitope. In fact, all of the hmAbs glycan profiling revealed a typical IgG1 glycoform distribution. hmAb3 displayed a distinctive glycan profile characterized by a markedly elevated abundance of highly galactosylated and sialylated glycoforms, such as G2F/G2F and G2F/G2FS1 as well as a non-negligible amount of nonfucosylated variants such as G2F/G2S1 and G2S1/G2FS1. Glycoforms were isolated by affinity chromatography and analyzed by the ADCC cellular assay. Notably, fraction 5 that showed the highest affinity for the FcγRIIIa in both affinity chromatography and cellular assays contained not only G2F/G2S1 as predominant glycan but also G2S1/G2FS1 and small amounts of G2S1/G2S1. Our findings suggest that hmAb3 can be considered a suitable candidate for developing therapeutic antibodies with broadly promiscuous, strong neutralizing, and ADCC activities. Additionally, glycoengineering processes could be considered for optimization of ADCC starting from recombinant hmAbs by increasing the content of afucosylated glycan with a high content of galactose and sialic acid. Additionally, the evaluation of glycoengineered mAbs suitable to stimulate other effector cells (i.e., macrophages or dendritic cells) could be evaluated to define the best approach for combining optimal neutralizing and cellular activity.

Summing up, this research not only offers valuable insights into the variability of glycosylation profiles among hmAbs produced by B-cells after vaccination or infection but also presents a strategic workflow for investigating the interaction between isolated glycoforms and the FcγRIIIa receptor, along with their functional evaluation by ADCC assays.

In conclusion, this multidisciplinary approach enables the correlation of specific glycoform patterns with functional relevance, providing a useful tool for the selection of glycoengineered therapeutic hmAbs with enhanced Fc-mediated activity. This approach has been investigated here for the selection of products that can provide the basis for the development of glycoengineered hmAbs with efficient neutralizing and ADCC activities targeting the SARS-CoV-2 virus or other emerging viral pathogens.

## 4 Experimental Section

### 4.1 Materials

Liquid chromatography–mass spectrometry (LC–MS) grade chemicals such as trifluoroacetic acid (TFA), ammonium acetate, ammonium formate, ammonium bicarbonate, formic acid, acetonitrile (ACN), ammonium hydroxide, 1,4-Dithiothreitol (DTT), Procainamide hydrochloride, and Sodium cyanoborohydride were purchased from Sigma-Aldrich (Milan, Italy). LC–MS grade IdeS and PNGase F enzymes were purchased from Promega (Milan, Italy). Milli-Q water was collected from a Direct-QTM Millipore system (Millipore, Billerica, MA, USA), and mobile phases were prepared in Milli-Q water. The pH of mobile phases was adjusted with acetic acid, formic acid, and ammonium hydroxide. Mobile phases were filtered using a 0.22 μm nylon membrane filter (Pall Life Sciences, NY, USA) and degassed before use. To generate hmAbs, the following reagents were used: Lympholyte (Cedarlane, Burlington, Canada), fetal bovine serum (FBS, Euroclone), dimethyl sulfoxide (DMSO, PanReac AppliChem), and PBS1X w/o Calcium and Magnesium (Biosigma). RPMI 1640 Dutch Modified (Biosigma) supplemented with 10% fetal calf serum (FCS, HyClone), 4 mM l-glutamine (Biosigma), 2 mM Na-Pyruvate (Biosigma), 1% nonessential amino acids (NEAA, Biosigma), 1% Penicillin/Streptomycin (Biosigma), IL-2 and IL-6 (Peprotech) 10 ng/mL, and oligonucleotide CpG-2006 (Mirosynt, CH) 3 μg/mL was used. Tween-20, 96-microplates, RPMI 1640 (VWR), Penicillin/Streptomycin, and Bovine serum albumin (BSA) were purchased from Fisher Scientific. HRP-conjugated Polyclonal Rabbit Anti-Human IgG and 3,3′, 5,5′-tetramethylbenzidine (TMB) peroxidase substrate were purchased from Prodotti Gianni S.p.A., and anti-CD107a FITC was purchased from BD Bioscience.

### 4.2 Wuhan and Omicron XBB.1.5 SARS-CoV-2 Spike RBD Cloning, Recombinant Production, and Labeling

The pCAGGS plasmid containing the sequence encoding the C-terminal His-tagged Wuhan SARS-CoV-2 Spike RBD (#NR_52310) was obtained from BEIRESOURCES (NY, USA). Variants were generated by replacing the SARS-CoV-2 Wuhan RBD-encoding sequence with synthetic sequences (Genewiz) encoding for either SARS-Cov-2 RBD Omicron and/or XBB.1.5 variants into the pCAGGS plasmid template using the 5′-XbaI and 3′-NotI restriction sites. Recombinant SARS-CoV-2 Spike RBDs were produced using polyethylenimine-based transient transfection of Freestyle HEK293 Cells (HEK293-F, Life Technologies) cultivated in suspension as described earlier.^48^ Purification was carried out as described in the literature.^49^ Briefly, cell media containing the secreted proteins of interest were collected 6 days after transfection by centrifugation at 1000*g* for 15 min. The pH and ionic strength of the filtered media were adjusted using concentrated phosphate-buffered saline (PBS). The samples were loaded onto a 5 mL His-Trap excel column (Cytiva) using a peristaltic pump and then eluted with a 3–250 mM imidazole gradient using an NGC fast protein liquid chromatography system (Bio-Rad). Peak fractions containing the antigens of interest were subjected to immediate concentration with concomitant buffer exchange with fresh PBS to remove imidazole using Amicon centrifugal filters (Merck). Quality controls during protein purification were carried out using reducing and nonreducing SDS-PAGE analysis and differential scanning fluorimetry with a Tycho NT.6 instrument (Nanotemper). All samples were concentrated to 1 mg/mL, flash-frozen in liquid nitrogen, and kept at −80 °C until usage.

Fluorescein labeling was carried out by incubating the recombinant RBD samples at 0.05 mg/mL with NHS-fluorescein (Thermo Fisher Scientific) dissolved in dimethyl sulfoxide (DMSO) in a 1:25 molar ratio in phosphate buffer saline (PBS) for 60 min at RT, followed by desalting using a PD-minitrap G-25 (Cytiva) equilibrated with PBS. Labeling efficiency was assessed by calculating the degree of labeling (DOL), defined as

$$DOL=\frac{{Abs}_{493}}{\varepsilon_{fluor}\times \left(\frac{{Abs}_{280}-\left({Abs}_{493}\times 0.3\right)}{\varepsilon_{protein}}\right)}$$

by measuring the sample absorbance using a NP80 (IMPLEN) microvolume spectrophotometer and using ε_fluor_ = 70′000 M^–1^·cm^–1^ according to manufacturer’s instructions, and ε_protein_ = 33′850 M^–1^·cm^–1^ as computed from Expasy ProtParam^50^ using the RBD sequence. For labeled RBD used in this work, DOL values were consistently higher than 0.8.

### 4.3 Human Monoclonal Antibodies Generation

Peripheral blood samples were collected from vaccinated or COVID-19-recovered subjects. All subjects signed informed consent. The study protocol conformed to the ethical guidelines of the 1975 Declaration of Helsinki and was approved by the Institutional Review Board and Ethical Committee of Fondazione IRCCS Policlinico San Matteo (document number 0047354/23).

PBMC were isolated by Lympholyte density gradient (1.077 g/mL) centrifugation according to the manufacturer’s instructions, frozen in a solution of 90% FBS and 10% DMSO, and stored in liquid nitrogen until use. Cryopreserved PBMC was then thawed and washed in PBS containing 2% FBS.

B lymphocyte enrichment was achieved by using the EasySep Human Pan-B Cell Enrichment Kit (STEMCELL Technologies) following the manufacturer’s instructions. This immune negative selection was followed by depletion of IgM+ cells: B lymphocytes were labeled with an anti-IgM fluorescein isothiocyanate (FITC, BD Bioscience) antibody. Subsequently, an anti-FITC antibody conjugated to magnetic particles (from EasySep Human FITC Positive Selection Kit II, STEMCELL Technologies) was added, allowing for the formation of an immunocomplex between the surface antibody expressed by the cells and the magnetic particles. Antigen-positive (Ag^+^) B lymphocytes were then isolated using the same anti-FITC magnetic separation protocol after labeling cells with the FITC-conjugated RBD (Wuhan or Omicron XBB.1.5) protein. Ag-specific B lymphocyte cultures were stabilized through transformation with the Epstein–Barr virus, resulting in the establishment of lymphoblastoid cell lines.

The Ag + B cells obtained were seeded on a feeder layer consisting of irradiated PBMC (3000 rad) in medium supplemented with FCS, oligonucleotide CpG-2006, and IL-2. After 5 days, IL-6 was added to further stimulate cell differentiation.

Cell growth was monitored daily, and wells that reached optimal expansion were screened using an indirect Enzyme-Linked Immunosorbent Assay (ELISA). Specifically, the RBD protein was coated onto 96-well microplates at a concentration of 0.5 μg/mL in 50 mM bicarbonate buffer (pH 9.6) and incubated at 4 °C for 48 h. After three washing steps with PBS containing 0.05% Tween-20, nonspecific binding sites were blocked by incubation with PBS supplemented with 2% BSA and 0.1% Tween-20 for 1 h. Subsequently, 100 μL of supernatant from the cell cultures was added and incubated at 37 °C for 2 h. Following three additional washing steps with PBS containing 0.05% Tween-20, the wells were incubated for 1 h at 37 °C with an appropriately diluted HRP-conjugated antihuman IgG secondary antibody (Dako). After a final washing step, the colorimetric reaction was developed using TMB (KPL, Seracare) as the substrate and absorbance were measured at 450 nm. Positive well cultures were cloned by successive limiting dilution at 50, 25, and 12.5 cells/well in medium w/o CpG until generation of monoclonal Ag-specific IgGs, as described previously.^51^ The ELISA assay was also employed to determine Ig subclasses using secondary antibodies specific for the γ1, γ2, γ3, and γ4 heavy chains (Invitrogen), as well as the κ (US Biological) and λ (Antibodies Online) light chains.

The three hmAbs obtained from B cell cloning were purified from the cell culture supernatant using affinity chromatography with a 1 mL MabSelect column (Cytiva). The loading buffer consisted of 20 mM sodium phosphate and 150 mM NaCl, pH 7.2, while elution was performed with 100 mM sodium citrate, pH 3.0. The pH of each 1 mL eluted fraction was immediately neutralized by adding 100 μL of 1 M Tris–HCl, pH 8.0. The fractions were analyzed by SDS-PAGE (Figure S1) using a 12% gel, and those containing antibody were pooled and subjected to buffer exchange using a HiPrep 26/10 desalting column (Cytiva) into cell culture-tested PBS. Eluted fractions were then pooled, concentrated using a 50 kDa molecular weight cutoff concentrator, and quantified by μBCA assay (Pierce), yielding approximately 20 mg/L.

### 4.4 Neutralization Assay

The antibody titers against SARS-CoV-2 Chinese-derived strain (Wuhan), the Italian strain (D614G), Beta strain (501Y.V2 lineage B.1.351), Delta strain (B.1.617.2), or Omicron strains (XBB.1.5 or EG.5) were measured by microneutralization assay. Briefly, 50 μL of serial 1:2 dilutions in three replicas, starting at 10 μg/mL to 0.07 μg/mL, of each B cell clone-derived mAbs was incubated with 50 μL of 100 TCID50/mL of SARS-CoV-2 VOCs for 1 h at 33 °C in a 5% CO_2_ atmosphere. After incubation, 3 × 10^4^ VERO E6 cells (VERO C1008 (Vero 76, cloneE6, Vero E6); ATCC CRL-1586) in 50 μL were added to each well, and the microplate was reincubated for an additional 72 h at the same conditions.

At the end of the incubation period, the supernatant was removed, and the plates were stained with Gram’s crystal violet solution (Merck KGaA, Darmstadt, Germany) plus 5% v/v formaldehyde 40% m/v (Carlo Erba SpA, Arese, Italy). Plates were read with the ELISA reader (Biochrom EZ read 400 microplate-reader), and data was analyzed using GraphPad Prism 8.3.0 (GraphPad Software, La Jolla, CA, USA). The concentration of hmAbs that produced the viral replication inhibition of at least 50% (IC50) is considered the minimum cutoff for neutralization activity. The concentration that inhibited at least 90% (IC90) is defined as a high neutralization activity.

All SARS-CoV-2 VOCs were isolated from infected patients as previously reported;^51^ all procedures involving SARS-CoV-2 VoCs were carried out at the Level 3 Biosafety Laboratory.

### 4.5 Reverse-Phase Liquid Chromatography Coupled with Mass Spectrometry (RPLC-MS)

Intact mass analysis of all three hmAb samples was performed by using a RPLC-MS method. Chromatographic separation was achieved on an AdvanceBio RP-mAb C4 superficially porous column (2.1 × 50 mm, 3.5 μm, 450 Å) (Agilent Technologies, USA) using an ExionLC AD UPLC system (Sciex, Framingham, MA, USA). The mobile phase consisted of 0.1% formic acid (FA) in Milli-Q water (mobile phase A) and 0.1% FA in ACN (mobile phase B). A 10 min linear gradient of mobile phase B (20–60% B) was applied at a flow rate of 0.3 mL/min, with the column temperature maintained at 60 °C. For mass spectrometric analysis, the UPLC system was directly coupled to a X500B QTOF system (Sciex) operating in the intact MS mode. The instrument parameters were set as follows: positive ionization mode, TOF-MS scan type, curtain gas at 45 arbitrary units (AU), ion source gases 1 and 2 at 50 psi, electrospray ionization (ESI) source temperature at 500 °C, ion spray voltage at 5000 V, time bins to sum at 120 AU, TOF scan range of 800–5000 *m*/*z*, accumulation time of 1 arbitrary unit, declustering potential at 250 V, collision energy at 15 V, and collision activated dissociation (CAD) gas at 7 AU. Data analysis was conducted using BioPharmaView software version 1.7 (Sciex).

### 4.6 Subunit Mass Analysis

Each hmAb sample (100 μg) was enzymatically digested with 100 units of IdeS in ammonium bicarbonate buffer (pH 7.3) to generate mAb subunits. The reaction mixture was incubated at 37 °C for 2 h, followed by reduction of disulfide linkages using 40 mM dithiothreitol (DTT) at 37 °C for 30 min. The resulting fragments were analyzed by using hydrophilic interaction liquid chromatography coupled with mass spectrometry (HILIC-MS). For HILIC separation, an AdvanceBio Glycan Mapping column (2.1 × 150 mm, 1.8 μm, 300 Å; Agilent Technologies, USA) was used on a Dionex UltiMate 3000 HPLC system (Thermo Scientific, San Jose, CA, USA). The mobile phase consisted of 0.1% TFA in Milli-Q water (mobile phase A) and 100% ACN with 0.1% TFA (mobile phase B). The separation was performed using a gradient elution: 85% to 70% B over 1.0 min, 70% to 65% B over 25 min, 60% to 15% B over 1.0 min for the column washing, and followed by 10 min of re-equilibration at 85% B. The flow rate was maintained at 0.4 mL/min, with the column temperature set to 35 °C. For mass spectrometric analysis, the HILIC chromatography was coupled to a Q Exactive Focus Orbitrap MS system (Thermo Scientific). The MS parameters were set as follows: sheath gas flow rate of 20 AU, auxiliary gas flow of 5 AU, and auxiliary gas temperature of 300 °C. The ESI source was operated at a spray voltage of 3.3 kV, with an inlet capillary temperature of 150 °C. An S-lens RF level of 50 V was applied. Data acquisition was performed in the *m*/*z* range of 500–3000 at a resolution of 70,000, with 1 microscan, an automatic gain control (AGC) target of 1 × 10^6^, and a maximum ion injection time of 50 ms. The system interfaces were operated by Xcalibur software (Thermo Scientific), while data processing was performed using the online UniDec software.^52^

### 4.7 Released N-Glycan Analysis

The hmAb samples were prepared for N-glycan analysis as previously described Segu.^53^ Briefly, 100 μg of hmAb was treated with 100 units of PNGase F enzyme in 25 mM phosphate-buffered saline (PBS), pH 7.2, and incubated overnight at 37 °C. Further, the samples were transferred to 30 kDa molecular weight cutoff filters (Amicon Ultra centrifugal filters, Sigma-Aldrich, Milan, Italy) with collection tubes and centrifuged at 14,000 rpm for 15 min. The glycan-containing flow-through was collected in Eppendorf tubes, and the procainamide labeling reagent was added in a 1:1 volume ratio (v/v). The mixture was incubated at 65 °C for 2–3 h. The labeling reagent was freshly prepared as previously described.^54^ Labeled glycans were purified using Hypercarb solid-phase extraction cartridges following the manufacturer’s protocol (Thermo Scientific). Purified glycans were analyzed using an AdvanceBio Glycan Mapping column (2.1 × 150 mm, 2.1 μm, 300 Å) (Agilent Technologies, USA) on a Dionex UltiMate 3000 HPLC system (Thermo Scientific). The mobile phases consisted of 50 mM ammonium formate, pH 4.5 (mobile phase A), and 100% acetonitrile (mobile phase B). The separation was performed using a gradient elution: 85% to 75% B over 3.0 min, 75% to 60% B over 45 min, 60% to 40% B over 1.0 min for the column washing, and followed by 10 min of re-equilibration at 85% B. The flow rate was maintained at 0.4 mL/min, with the column temperature set to 35 °C. For mass spectrometric analysis, the HILIC chromatography was coupled to a Q Exactive Focus Orbitrap MS system (Thermo Scientific). The MS parameters were set as follows: sheath gas flow rate of 20 AU, auxiliary gas flow of 5 AU, and auxiliary gas temperature of 300 °C. The ESI source was operated at a spray voltage of 3.5 kV, with an inlet capillary temperature of 275 °C. An S-lens RF level of 50 V was applied. Data acquisition was performed in the *m*/*z* range of 500–3000 at a resolution of 70,000, with 1 microscan, an AGC target of 3 × 10^6^, and a maximum ion injection time of 100 ms. The system interfaces were operated by Xcalibur software (Thermo Scientific).

### 4.8 FcyRIIIa Affinity Chromatography

For the separation of functional glycoforms, three hmAb samples were analyzed using a TSKgel FcR-IIIa-NPR column (4.6 × 75.0 mm; Tosoh Bioscience, Darmstadt, Germany) on an ExionLC AD UPLC system (Sciex, Framingham, MA, USA). The mobile phases consisted of 50 mM ammonium acetate (pH 6.8) (mobile phase A) and 50 mM ammonium acetate (pH 4.5) (mobile phase B). Chromatographic separation was achieved using a linear gradient of 100% B over 30 min at a flow rate of 0.35 mL/min, with the column maintained at 25 °C. For native MS analysis, the UPLC system was directly coupled to a X500B QTOF system (Sciex, Framingham, MA, USA) operating in the intact MS mode. The instrument parameters were set as follows: positive ionization mode, TOF-MS scan type, curtain gas at 40 AU, ion source gases 1 and 2 at 40 psi, electrospray ionization (ESI) source temperature at 350 °C, ion spray voltage at 5000 V, time bins to sum at 120 AU, TOF scan range of 5000–10,000 *m*/*z*, accumulation time of 1 arbitrary unit, declustering potential at 100 V, collision energy at 5 V, and CAD gas at 5 AU. Data analysis was conducted using BioPharmaView software version 3.0 (Sciex, Framingham, MA, USA). For the glycosylation analysis, hmAb samples resolved through FcγRIIIa affinity chromatography were collected, and intact mass analysis was performed for the collected fractions as mentioned above. The collected fractions were enzymatically deglycosylated using PNGase F to determine the molecular masses of the glycan-free proteins. For deglycosylation, 20 μg of each fraction was treated with 20 Units of PNGase F enzyme, and the reaction mixture was incubated at 37 °C for overnight. The deglycosylated samples were subsequently analyzed by using the same RP-LC method. Data analysis was conducted using the BioPharmaView software 1.7 (Sciex).

### 4.9 Antibody-Dependent Cell-Mediated Cytotoxicity Assay

An ADCC surrogate assay was employed to evaluate effector cell activation directly mediated by the interaction between antibody Fc regions and Fcγ receptors rather than cytotoxic effects on target cells. In this system, effector cell activity was assessed in the absence of target cells. Briefly, 100 μL/well of purified hmAbs at different concentrations (5, 10, 20, and 40 μg/mL) in PBS was coated onto 96-well plates (Immuno Clear Standard Modules, Thermo Fisher Scientific) and incubated at 4 °C overnight. After washing with PBS containing 2% FCS, previously isolated PBMCs from a single healthy donor recruited through our blood donation center were seeded at 3 × 10^5^ cells/well in culture medium containing CD107a-FITC (BD Biosciences). Following a 4 h incubation at 37 °C in a humidified atmosphere with 5% CO_2_, cells were harvested, washed with PBS containing 2% FCS, and stained with anti-CD3 BV510 and anti-CD56 BB700 antibodies (both from BD Biosciences) at 4 °C for 30 min. After a final wash with PBS 2% FCS, cells were fixed. Data acquisition and analysis were performed using a FACS Celesta flow cytometer (BD Biosciences) and Kaluza software (Beckman Coulter).

## Supplementary Material



## Notes

SDS-PAGE and Coomassie staining of hmAbs, intact mass analysis of anti-SARS-CoV-2 hmAbs, intact mass analysis of deglycosylated anti-SARS-CoV-2 hmAbs, subunit analysis of all three hmAbs, glycoform assignments of each peak of hmAbs, degranulating activity of NK cells mediated by several concentrations by 10 μg/mL of the hmAb3-resolved fractions, and released N-Glycan analysis of all three hmAbs (PDF)

## Abbreviations Used

Abs: antibodies
ACN: acetonitrile
ADCC: antibody-dependent cellular cytotoxicity
AGC: automatic gain control
CHO: Chinese Hamster Ovary
CID: collision activated dissociation
COVID-19: coronavirus disease 2019
DMSO: dimethyl sulfoxide
DSF: differential scanning fluorimetry
FA: formic acid
FBS: fetal bovine serum
Fc: crystallizable fragment
FcγRs: Fcγ receptors
FITC: fluorescein isothiocyanate
Gal: galactose
GlcNAc: N-acetylglucosamine
HILIC-MS: hydrophilic interaction liquid chromatography–mass spectrometry
hmAbs: human monoclonal antibodies
ITAM: immunoreceptor tyrosine-based activation motif
Kda: kilodalton
LC: light chain
mAbs: monoclonal antibodies
Neu5Ac: sialic acid
NK: natural killer
PBMC: peripheral blood mononuclear cells
PTMs: post-translational modifications
RBD: receptor-binding domain
RP-LC: reverse-phase liquid chromatography
SARS-CoV-2: severe acute respiratory syndrome coronavirus-2
SPE: solid-phase extraction
TIC: total ion chromatogram
TMB: 3,3′, 5, 5′- tetramethylbenzidine
VoC: variants of concern
WHO: world health organization.
