# Supplementary material for: Selection of Functional Glycoforms in Anti-SARS-CoV‑2 Human IgG1 Monoclonal Antibodies by FcγRIIIa Affinity Chromatography and Mass Spectrometry
Source: J Med Chem. 2026 Apr 4;69(8):9441–56. doi: 10.1021/acs.jmedchem.6c00166 (PMC13126678; doi:10.1021/acs.jmedchem.6c00166)
Supplement: Supplementary file 1 [file jm6c00166_si_001.pdf]

Supporting Information

Selection of functional glycoforms in anti-SARS-CoV-2 Human IgG1 Monoclonal Antibodies by FcγRIIIa Affinity-Chromatography and Mass Spectrometry

Barbara Oliviero[1**], Sunil Kumar[2**], Daniela Conteianni[1], Gaia Donetti[1], Antonella Cerino[1], Antonino Samuele Iraci[1], Alessia La Gaipa[1], Sabrina Ottolini[1], Sara Tengattini[2], Gabriella Massolini[2], Irene Cassaniti[3,4], Josè Camilla Sammartino[3], Dalila Mele[4], Fausto Baldanti[3,4], Federico Forneris[5], Silvia Faravelli[5], Claudia Scotti[6], Greta Pessino[6], Maristella Maggi[6], Stefania Mantovani[1*], Caterina Temporini[2*], Mario U. Mondelli[1,7+], Marco Terreni[2+].

[1] Department of Translational and Clinical Research, Division of Molecular Medicine, Laboratory of Clinical Immunology, Fondazione IRCCS Policlinico San Matteo, 27100 Pavia, Italy.

[2] Department of Drug Science, University of Pavia, Pavia 27100, ITALY

[3] Department of Clinical-Surgical, Diagnostic and Pediatric Sciences, Università degli Studi di Pavia, Pavia, Italy

[4] Department of Microbiology and Virology, Fondazione IRCCS Policlinico San Matteo, 27100 Pavia, Italy.

[5] Department of Biology and Biotechnology, The Armenise-Harvard Laboratory of Structural Biology, University of Pavia, Pavia, Italy.

[6] Department of Molecular Medicine, Unit of Immunology and General Pathology, University of Pavia, Pavia, Italy.

[7] Department of Internal Medicine and Therapeutics, University of Pavia, Pavia, Italy.

** These authors contributed equally to the work

+ These authors share last authorship

* Corresponence:

Caterina Temporini

caterina.temporini@unipv.it

Stefania Mantovani

s.mantovani@smatteo.pv.it

Contents of SI

Figure S1: SDS-PAGE and Coomassie staining of hmAbs	S3
Figure S2A: Intact mass analysis of Anti-SARS-CoV-2 Human Monoclonal Antibodies	S4
Figure S2B: Intact mass analysis of de-glycosylated Anti-SARS-CoV-2 Human Monoclonal Antibodies	S5
Figure S3: Subunit analysis of hmAbs	S6-S7
Figure S4: Glycoform assignments of each peak of hmAbs	S8-S13
Figure S5: Degranulating activity of NK cells mediated by several concentrations by 10 ug/ml of the hmAb3 resolved fractions	S14
Table S1: Released N-Glycan labelled with Procainamide and determined the identification by HILIC-MS	S14

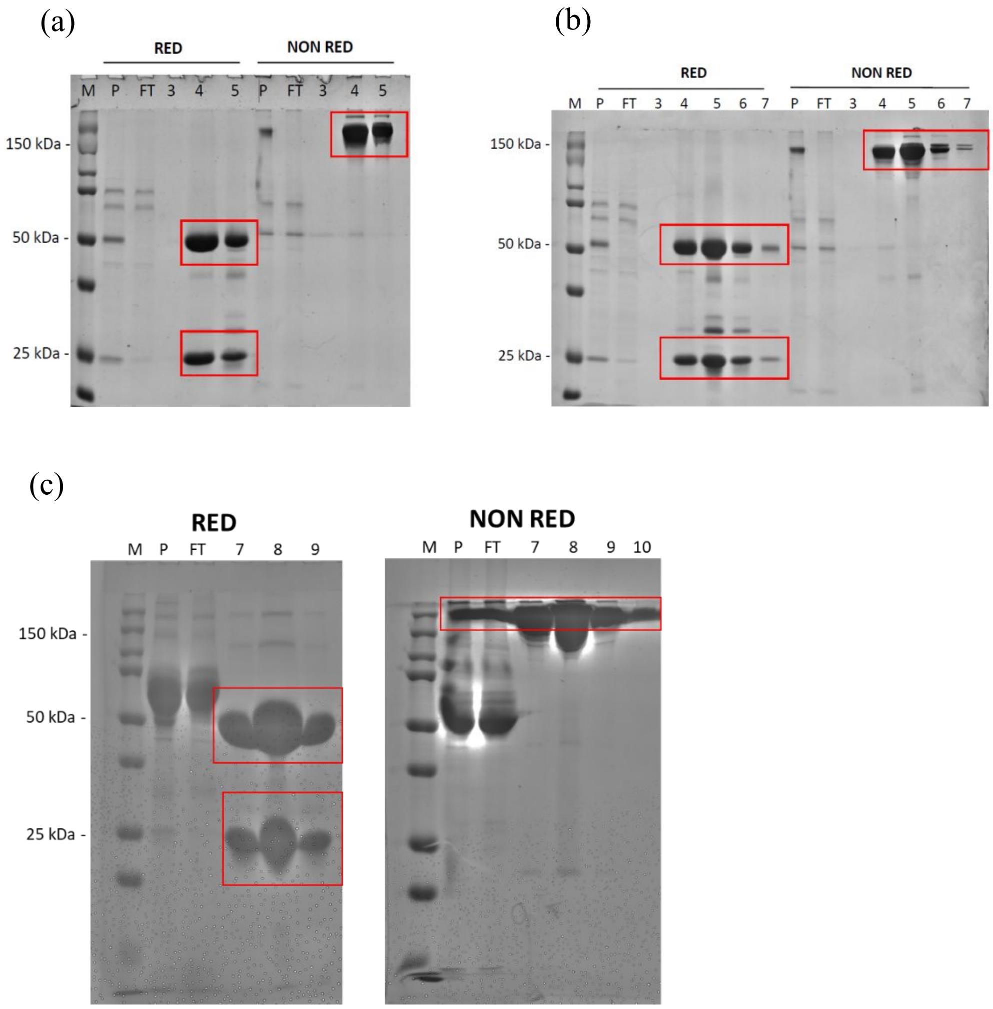

Figure S1: SDS-PAGE and Coomassie staining of hmAb1 (a), hmAb2 (b) in reducing (RED) and non-reducing conditions (NON-RED); (c) hmAb3 in non-reducing (left panel) and reducing conditions (right panel). M: Marker (Dual precision protein standard, BioRad); P: Pre-loading; FT: Flow-through. Numbers refer to the elution fractions.

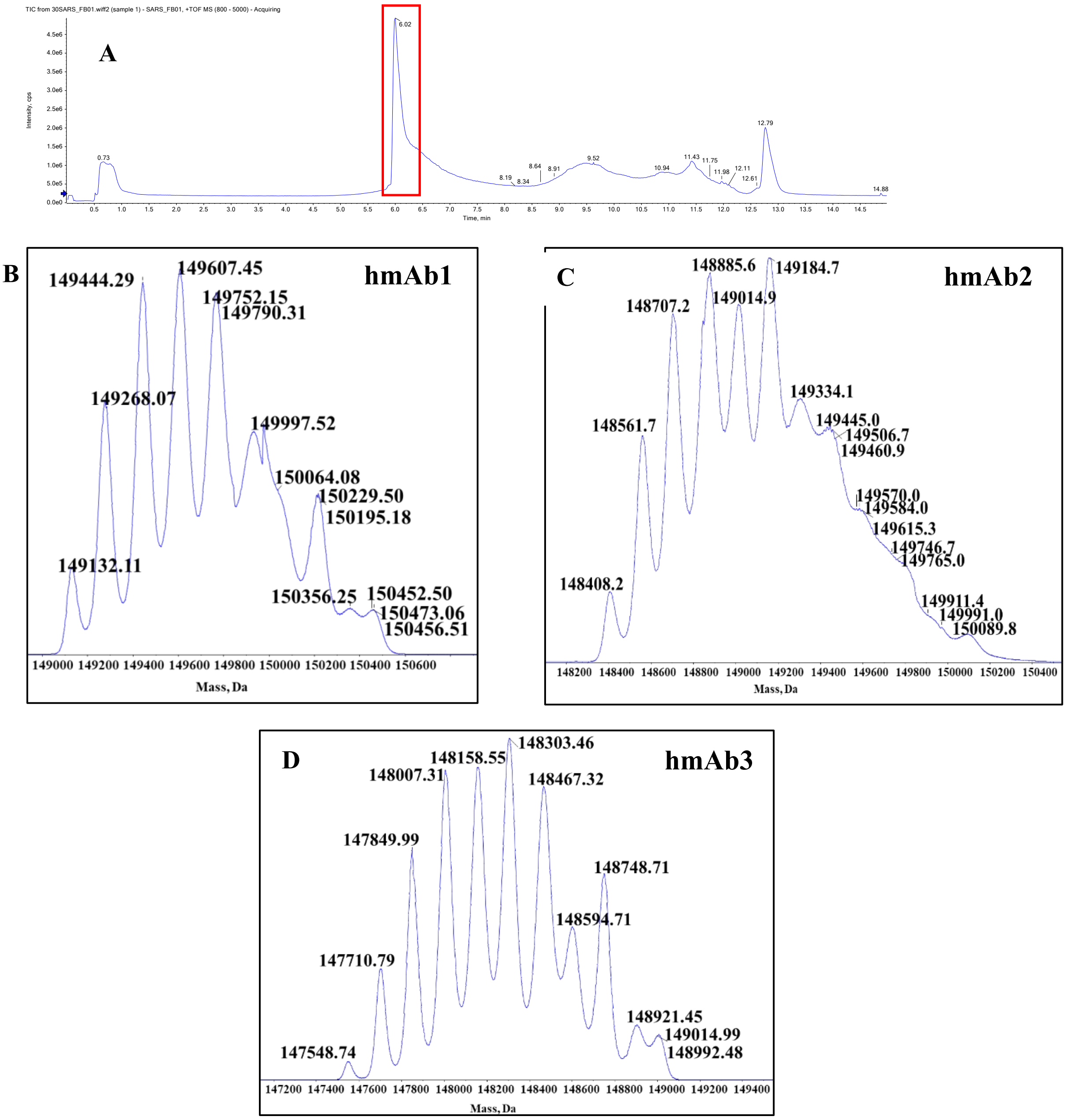

Figure S2A: Intact mass analysis of Anti-SARS-CoV-2 Human Monoclonal Antibodies; A: Total ion chromatogram of hmAb sample using RP-MS; B-D: Deconvoluted molecular mass result of hmAb1-3

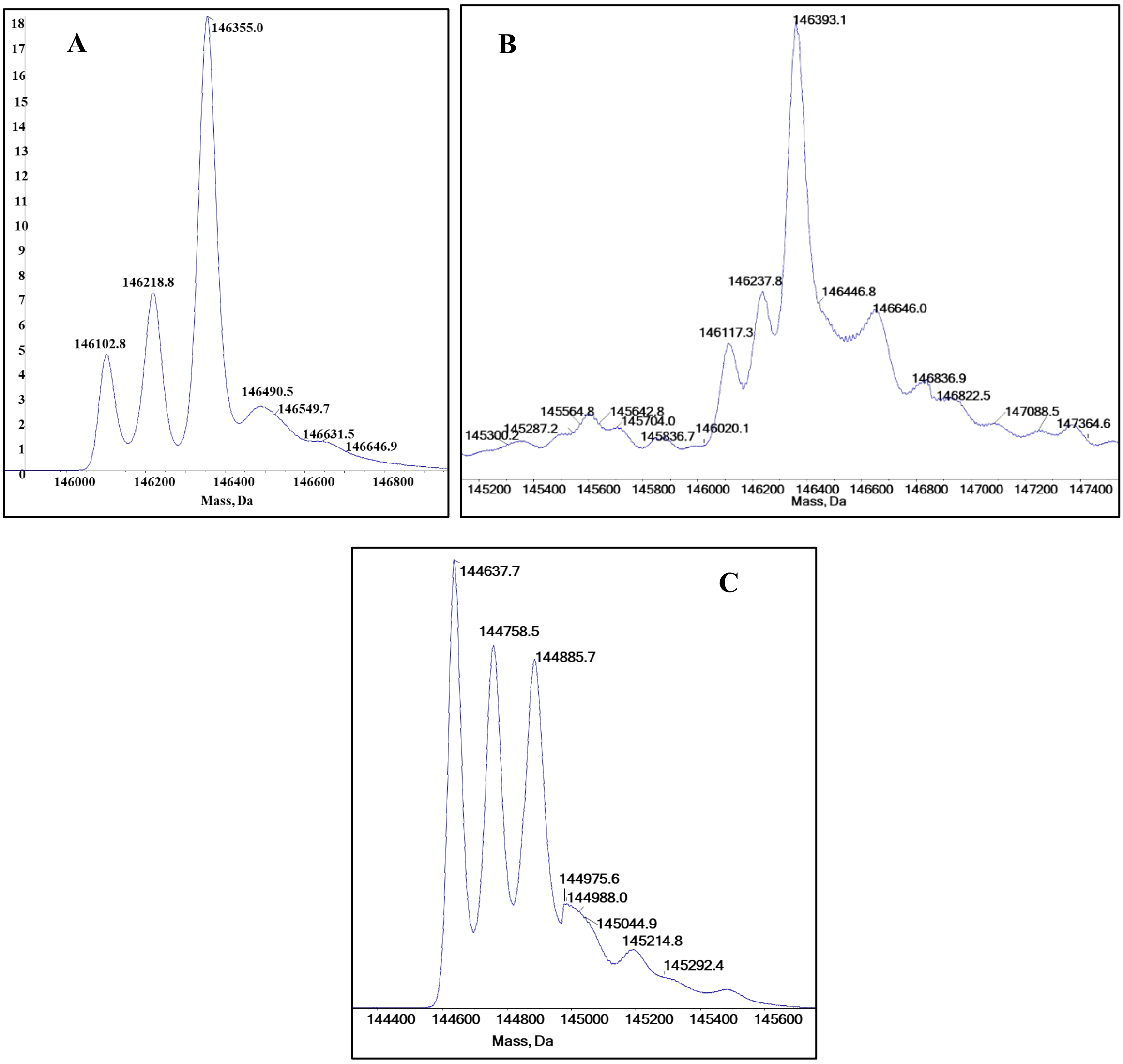

Figure S2B: Intact mass analysis of de-glycosylated Anti-SARS-CoV-2 Human Monoclonal Antibodies; A-C: Deconvoluted molecular mass result of De-glycosylated hmAb1-3

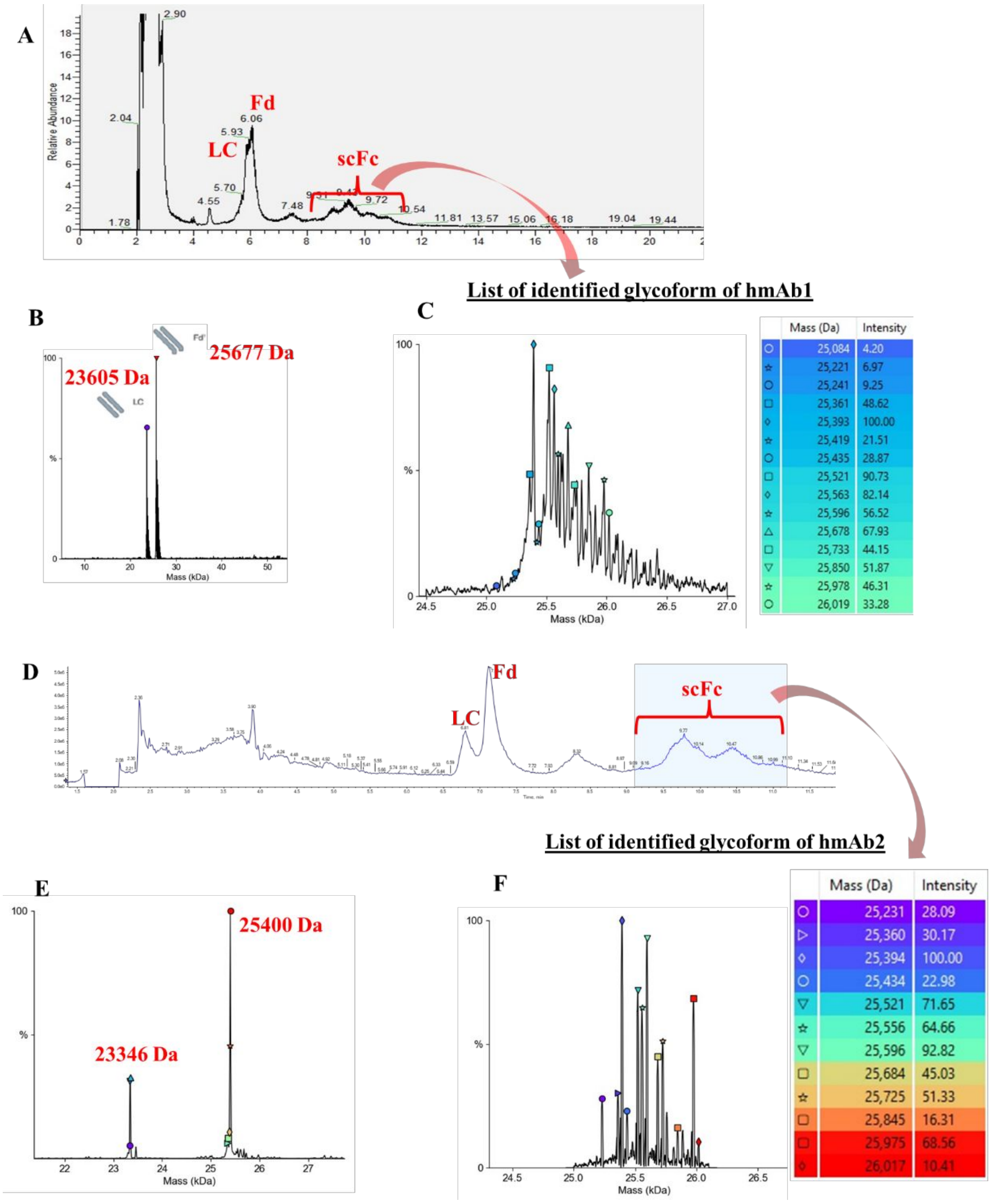

List of identified glycoform of hmAb1

Mass (Da)	Intensity
25,084	4.20
25,221	6.97
25,241	9.25
25,361	48.62
25,393	100.00
25,419	21.51
25,435	28.87
25,521	90.73
25,563	82.14
25,596	56.52
25,678	67.93
25,733	44.15
25,850	51.87
25,978	46.31
26,019	33.28

List of identified glycoform of hmAb2

Mass (Da)	Intensity
25,231	28.09
25,360	30.17
25,394	100.00
25,434	22.98
25,521	71.65
25,556	64.66
25,596	92.82
25,684	45.03
25,725	51.33
25,845	16.31
25,975	68.56
26,017	10.41

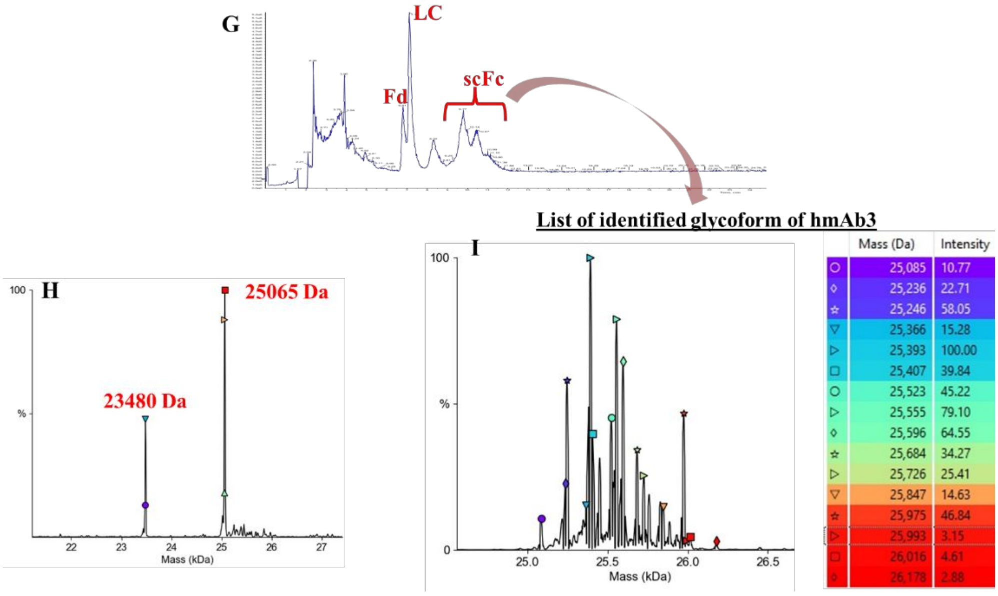

Figure S3: Subunit analysis of hmAbs. (A, D, G) Total ion chromatograms of the subunits from hmAb1–3 samples; (B, E, H) Deconvoluted mass spectra of the light chain (LC) and Fd′ fragments; (C, F, I) Deconvoluted mass spectra of the single-chain Fc (scFc) fragments.

Identification of glycoforms: Glycoform assignments were performed by analyzing each FcγRIIIa affinity-purified fraction of the hmAbs in both glycosylated and deglycosylated forms. The glycoforms were identified based on the mass differences of the major peaks, as illustrated in Fraction 01 of hmAb1. See panels in **Figure S4** below:

<u>**Intact molecular mass of Fraction 01 of hmAb1**</u>

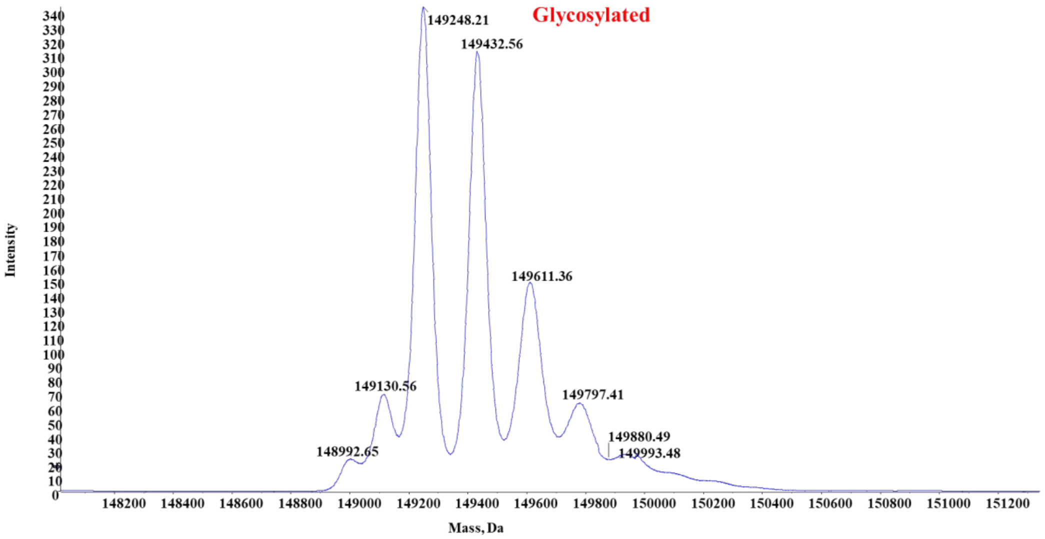

<u>**hmAb1 Fraction 01 (Treated with PNGaseF)**</u>

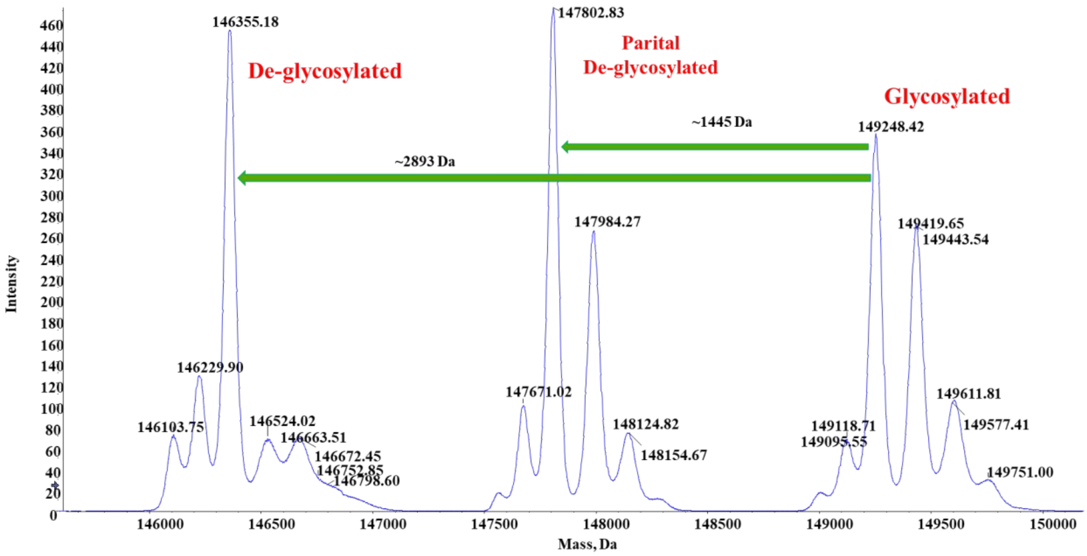

hmAb1 _Fraction 02 (Treated with PNGaseF)

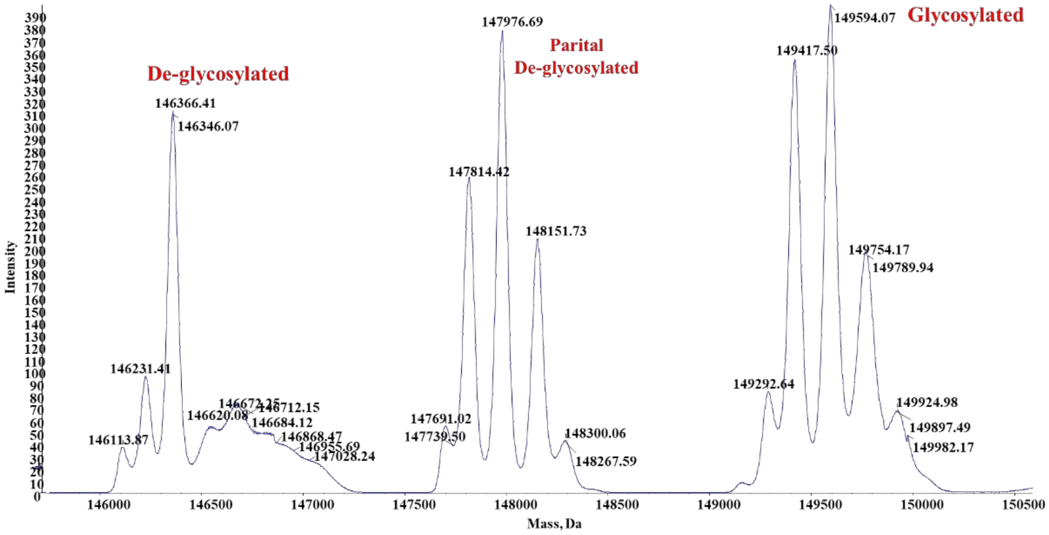

hmAb1 Fraction 03 (Treated with PNGaseF)

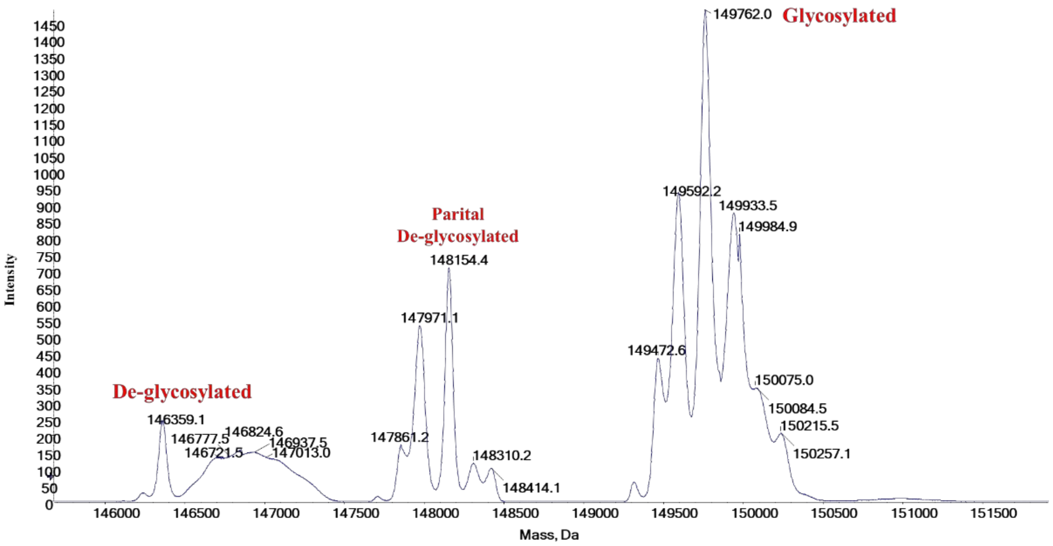

hmAb1 Fraction 04 (Treated with PNGaseF)

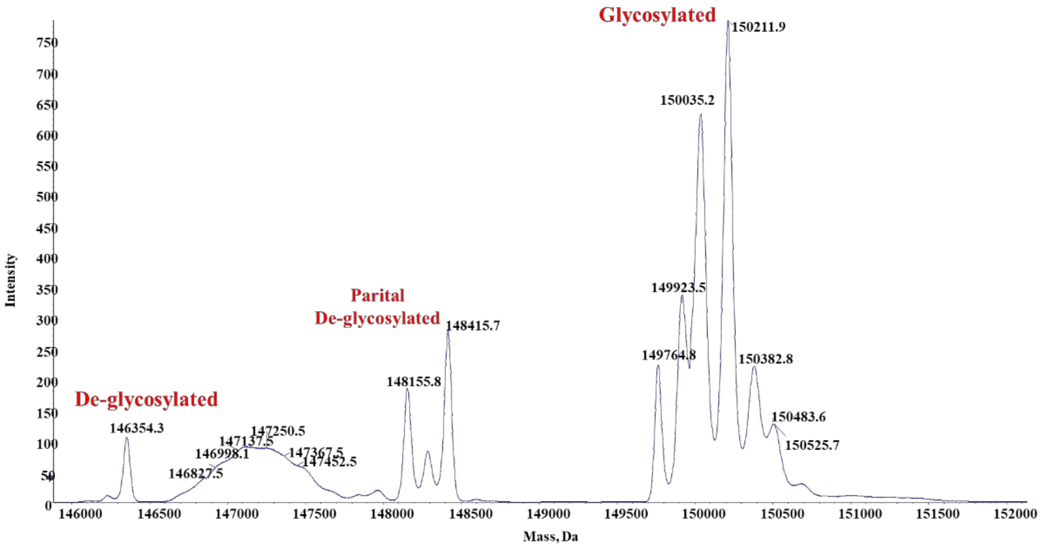

hmAb1 _ Fraction 05 (Treated with PNGaseF)

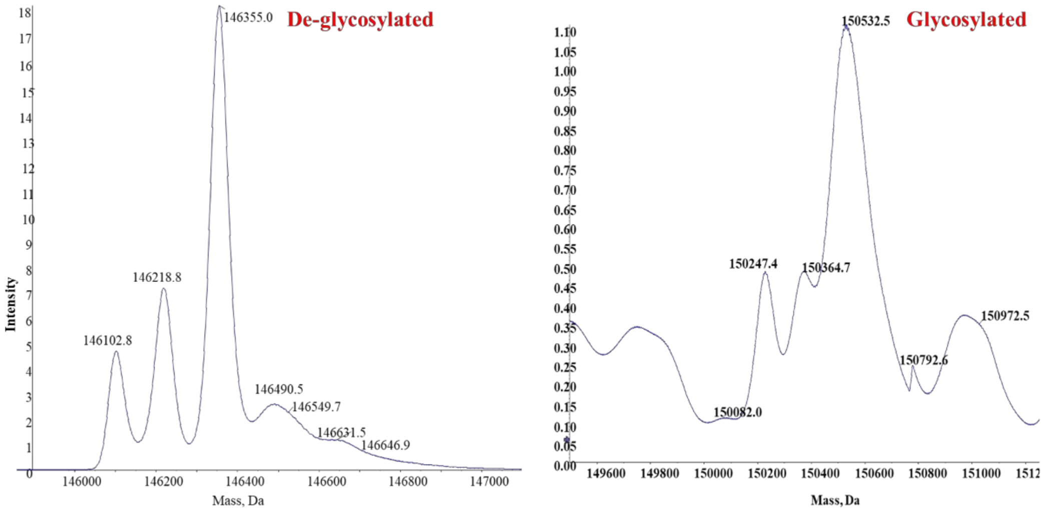

hmAb2 Fraction 01 (Treated with PNGaseF)

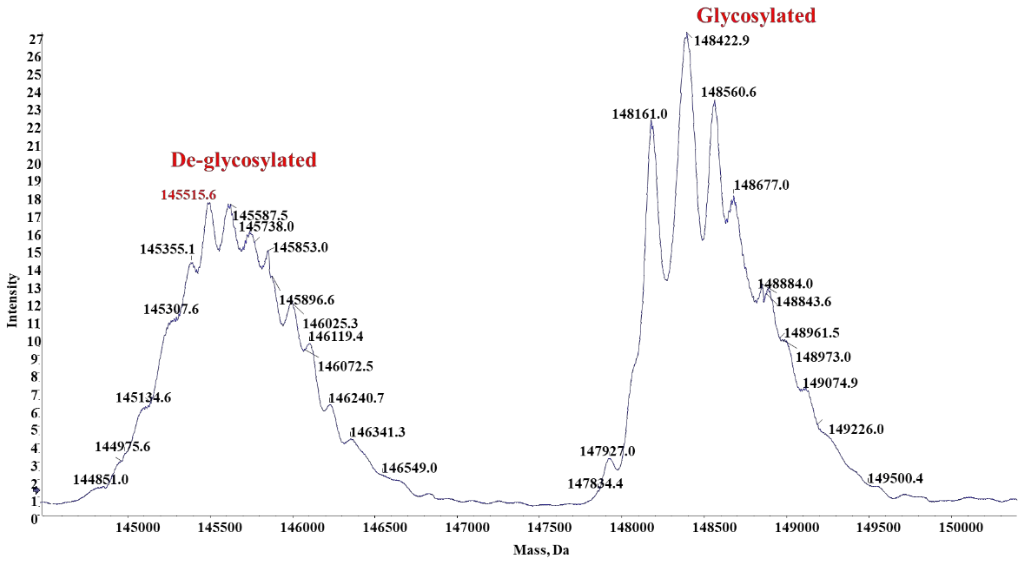

hmAb2 Fraction 02 (Treated with PNGaseF)

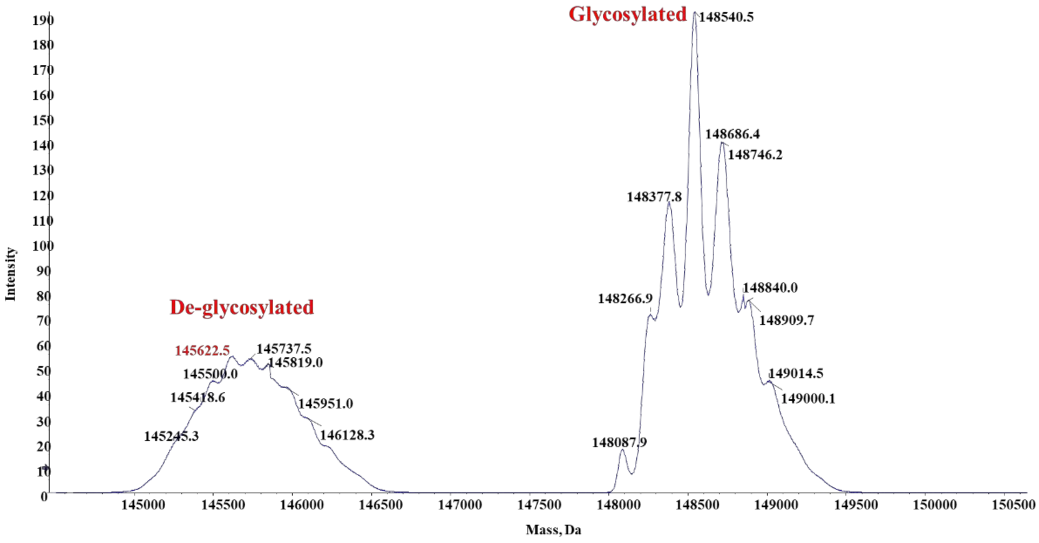

hmAb2 Fraction 03 (Treated with PNGaseF)

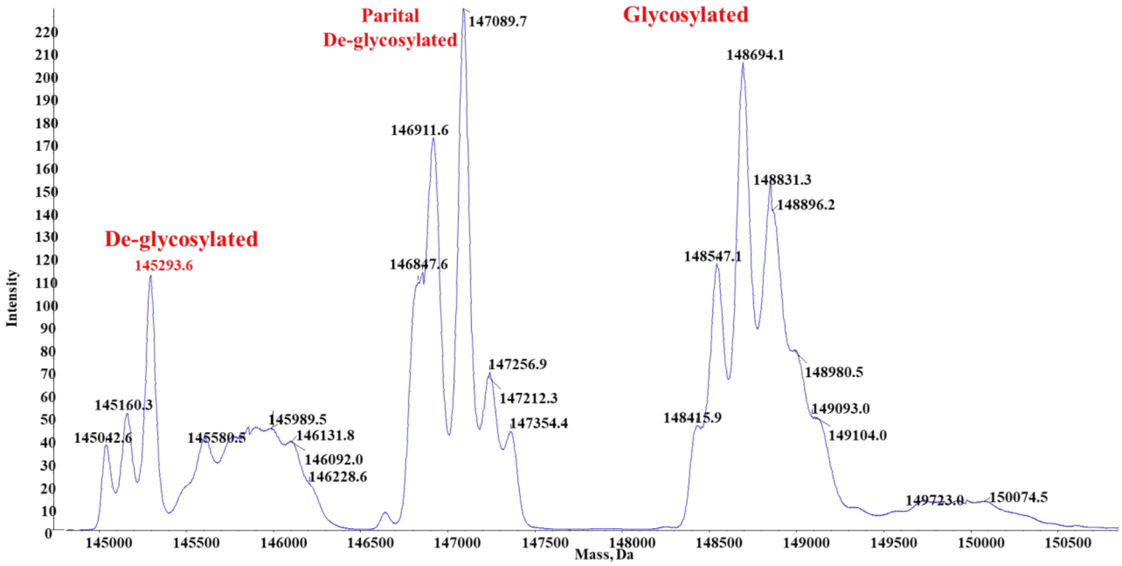

hmAb2 Fraction 04 (Treated with PNGaseF)

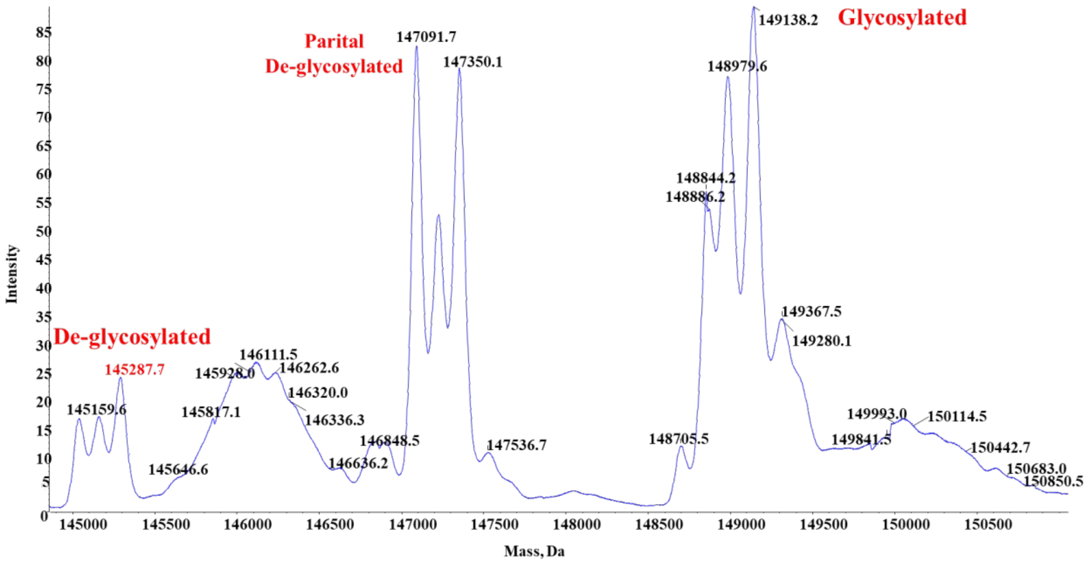

hmAb2_Fraction 05 (Treated with PNGaseF)

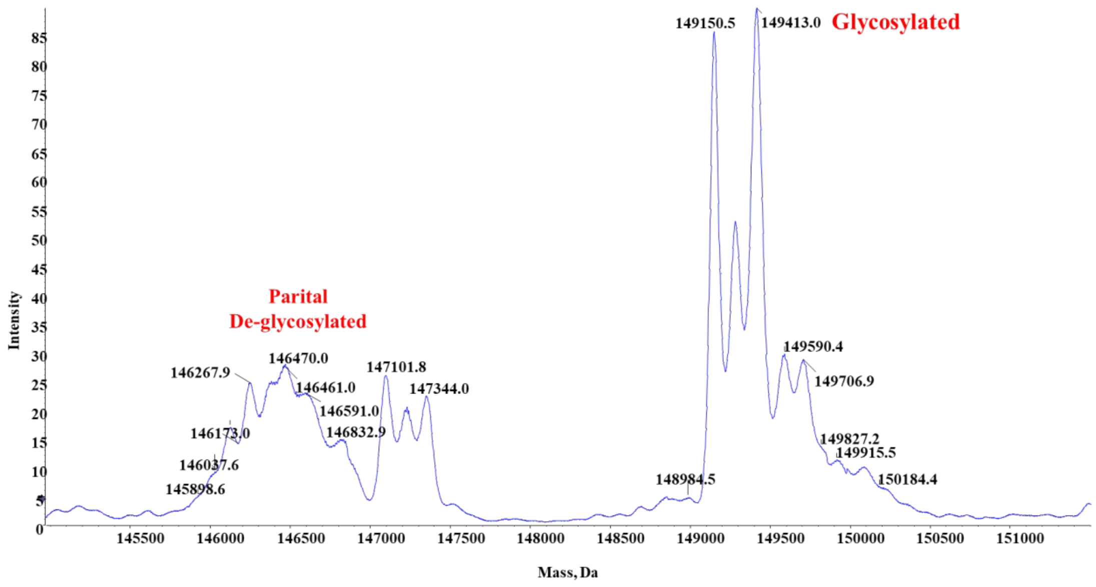

hmAb3_Fraction 01 (Treated with PNGaseF)

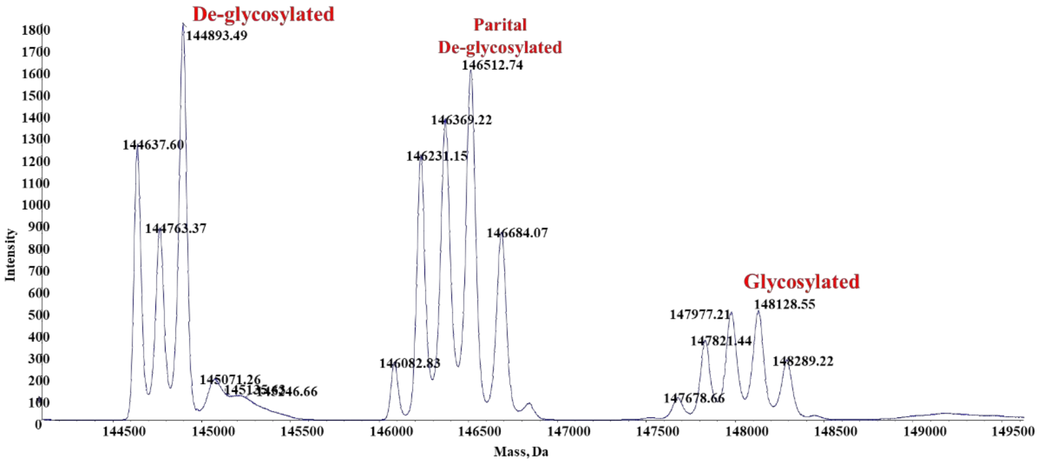

hmAb3_Fraction 02 (Treated with PNGaseF)

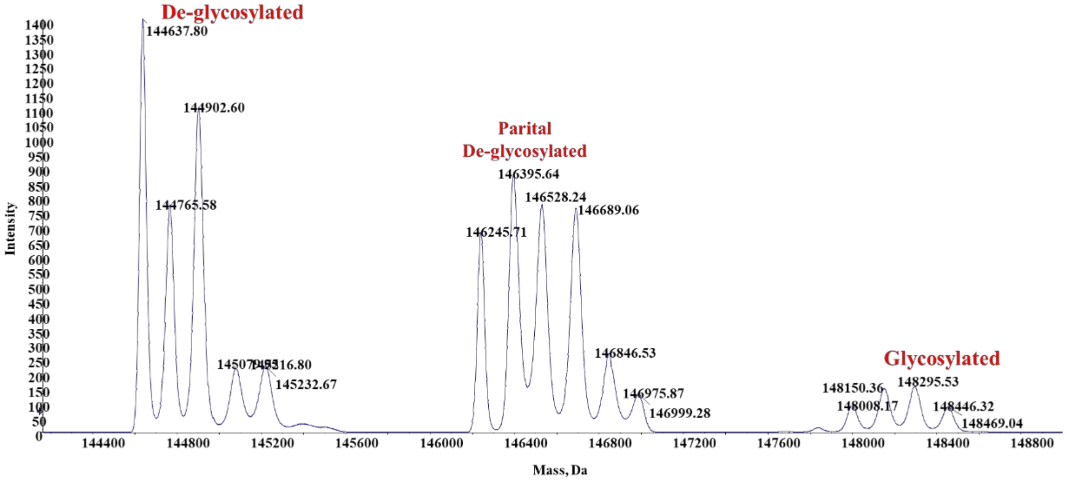

hmAb3 Fraction 03 (Treated with PNGaseF)

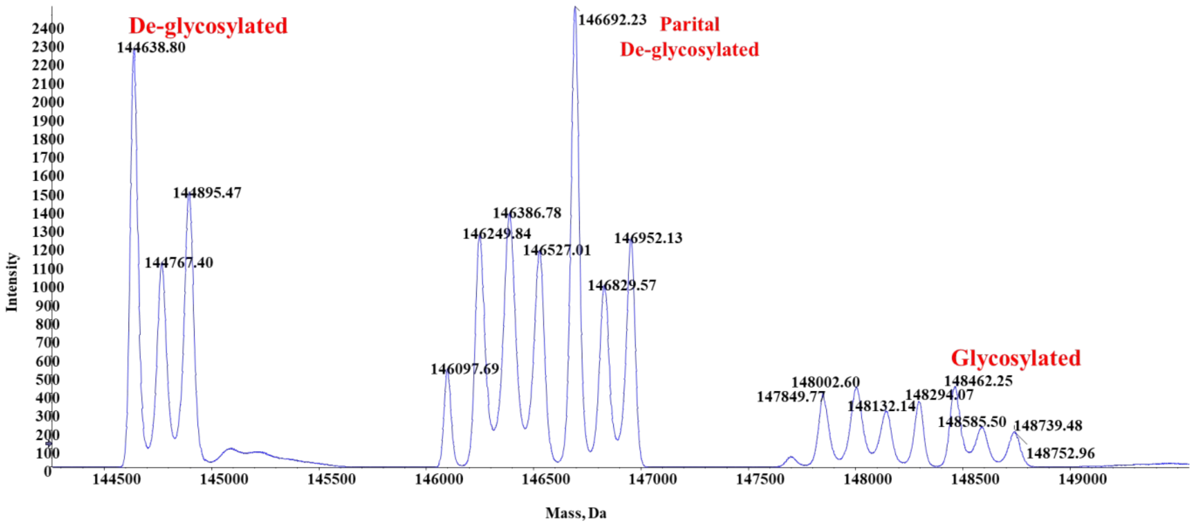

hmAb3 Fraction 04 (Treated with PNGaseF)

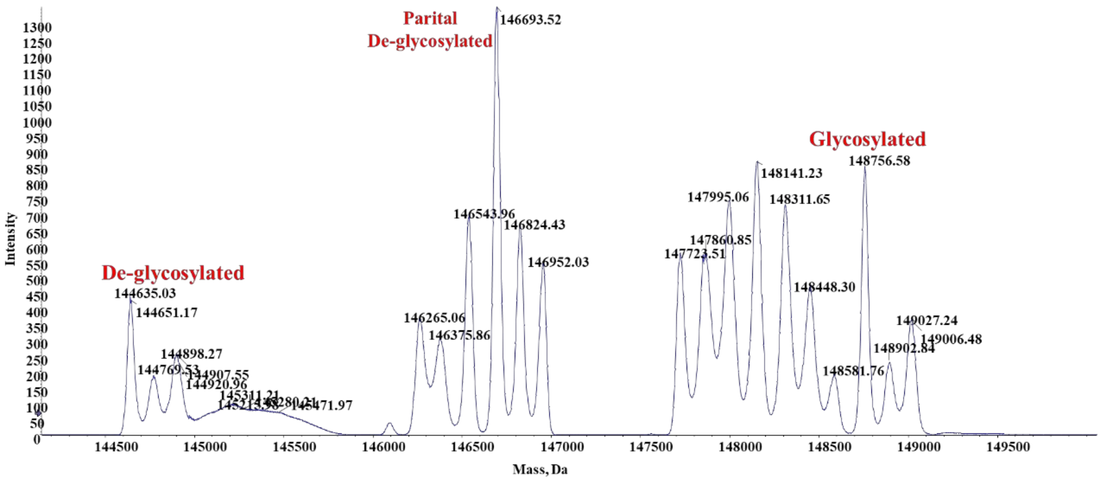

hmAb3 Fraction 05 (Treated with PNGaseF)

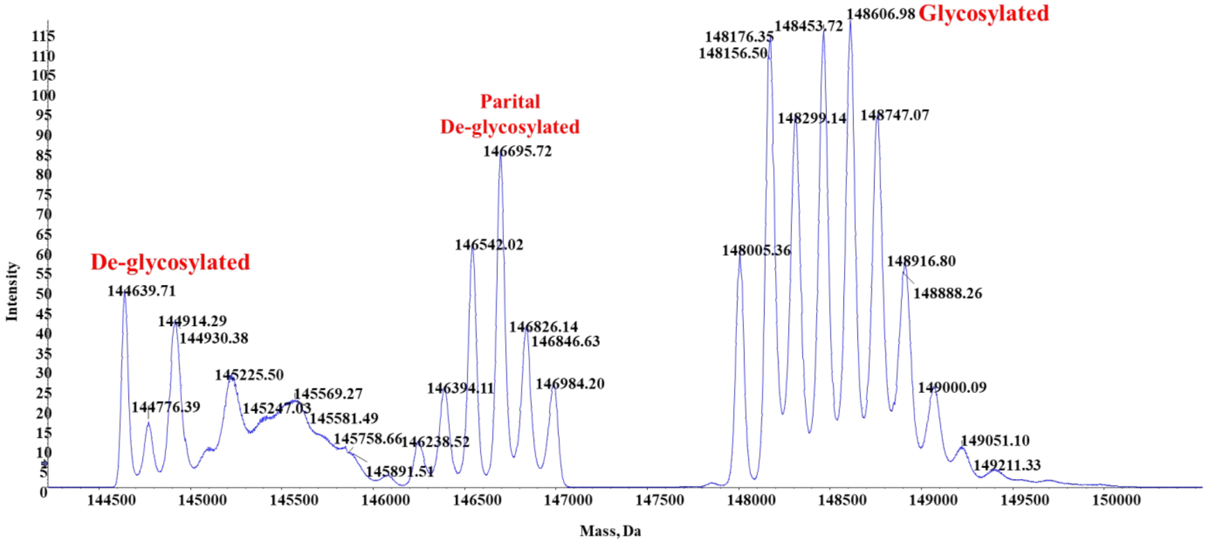

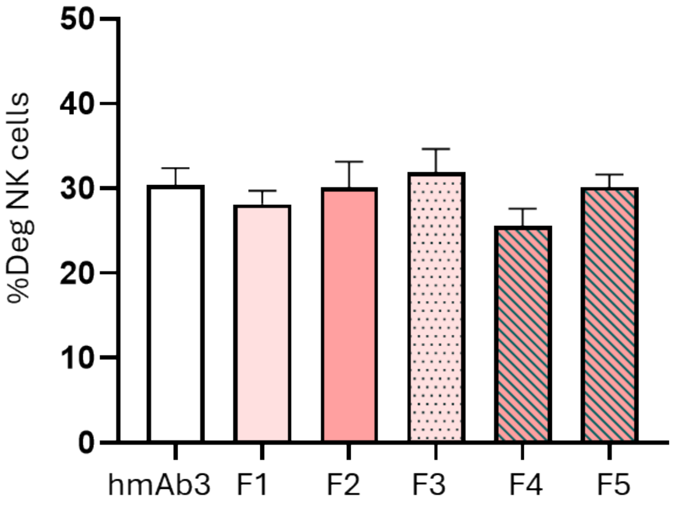

Figure S5: Degranulating activity of NK cells mediated by several concentrations by 10 ug/ml of the hmAb3 resolved fractions (F1-5).

Table S1: Released N-Glycan labelled with Procainamide and determined the identification by HILIC-MS

**Glycan**	**Procainamide labeled Glycan (*m/z*)**	**Relative abundance (%)**		
		**hmAb1**	**hmAb2**	**hmAb3**
G0F-N	1479.65	1.50	ND*	ND*
G0	1536.67	ND*	ND*	4.81
G0B	1739.75	ND*	1.89	6.11
G0F	1682.73	33.97	13.54	
G0FB	1885.81	7.66	10.35	1.95
Man5	1454.62	1.10	2.03	ND*
G1	1698.73	ND*	6.08	14.59
G1 + Na Adduct	1720.71	ND*	ND*	
G1B	1901.80	ND*	ND*	6.11
G1B + Na Adduct	1923.79	ND*	ND*	
G1F	1844.78	29.15	23.04	16.75
G1FB	2047.86	11.11	21.13	21.45
G1FS1	2135.88	2.11	ND*	ND*
Man6	1616.67	2.04	1.65	1.01
G2	1860.78	ND*	ND*	2.09
G2S1	2151.87	ND*	ND*	3.06
G2B	2063.86	ND*	ND*	0.43
G2F	2006.83	7.99	12.56	13.41
G2FS1	2298.93	2.26	6.39	4.19
G2FB	2210.92	1.12	1.33	1.96
G2FS1B	2501.02	ND*	ND*	2.07

*ND: Not detectable
